# Bat and pangolin coronavirus spike glycoprotein structures provide insights into SARS-CoV-2 evolution

**DOI:** 10.1038/s41467-021-21767-3

**Published:** 2021-03-11

**Authors:** Shuyuan Zhang, Shuyuan Qiao, Jinfang Yu, Jianwei Zeng, Sisi Shan, Long Tian, Jun Lan, Linqi Zhang, Xinquan Wang

**Affiliations:** 1 The Ministry of Education Key Laboratory of Protein Science, Beijing Advanced Innovation Center for Structural Biology, Beijing Frontier Research Center for Biological Structure, Collaborative Innovation Center for Biotherapy, School of Life Sciences, Tsinghua University, 100084 Beijing, China; 2 Center for Global Health and Infectious Diseases, Comprehensive AIDS Research Center, Beijing Advanced Innovation Center for Structural Biology, School of Medicine, Tsinghua University, Beijing, China

**Keywords:** Electron microscopy, SARS-CoV-2, Cryoelectron microscopy

## Abstract

In recognizing the host cellular receptor and mediating fusion of virus and cell membranes, the spike (S) glycoprotein of coronaviruses is the most critical viral protein for cross-species transmission and infection. Here we determined the cryo-EM structures of the spikes from bat (RaTG13) and pangolin (PCoV_GX) coronaviruses, which are closely related to SARS-CoV-2. All three receptor-binding domains (RBDs) of these two spike trimers are in the “down” conformation, indicating they are more prone to adopt the receptor-binding inactive state. However, we found that the PCoV_GX, but not the RaTG13, spike is comparable to the SARS-CoV-2 spike in binding the human ACE2 receptor and supporting pseudovirus cell entry. We further identified critical residues in the RBD underlying different activities of the RaTG13 and PCoV_GX/SARS-CoV-2 spikes. These results collectively indicate that tight RBD–ACE2 binding and efficient RBD conformational sampling are required for the evolution of SARS-CoV-2 to gain highly efficient infection.

## Abstract (web summary)

The spike glycoprotein in coronaviruses is a key viral protein for cross-species transmission and infection. Here, the authors present the cryo-EM structures of the spike ectodomains from bat and pangolin coronaviruses, compare them with the available SARS-CoV-2 spike structures and discuss implications for the evolution and cross-species transmission of SARS-CoV-2.

## Introduction

Zoonotic transmission of novel coronaviruses has been posing a tremendous threat to human health, as evidenced by the emergence of SARS-CoV in 2002–2003, MERS-CoV in 2012 and SARS-CoV-2 since the end of 2019^1–5^. SARS-CoV-2 is responsible for the ongoing global COVID-19 pandemic, which has caused more than one hundred million of infections and two million of deaths worldwide (https://covid19.who.int/). Current data suggest that like SARS-CoV and MERS-CoV^6^, SARS-CoV-2 likely originated in bats and eventually spread to humans following evolution in intermediate hosts. Coronavirus RaTG13, detected in the horseshoe bat *Rhinolophus affinis* in China’s Yunnan province, was identified as the closest relative of the SARS-CoV-2^5^. It shares 96.2% sequence identity with the SARS-CoV-2 genome, reflecting the likely origin of SARS-CoV-2 in bats^5^. Pangolin coronaviruses (PCoV) closely related to SARS-CoV-2 have also been identified in smuggled Malayan pangolins (*Manis javanica*) in China’s Guangxi (GX) and Guangdong (GD) provinces. Analyses of six PCoV_GX and four PCoV_GD genome sequences indicated a high level of similarity with SARS-CoV-2 (85.5% to 92.4% sequence identity)^7–10^. The natural reservoirs and intermediate hosts of SARS-CoV-2 remain a topic of debate, and it is still unclear how SARS-CoV-2 evolved to infect humans.

The homotrimeric spike (S) glycoprotein of coronaviruses forms a trimer, which plays a critical role in host cell attachment and entry by recognizing cellular receptors and mediating membrane fusion. Consequently, the spike protein, particularly its receptor-binding domain (RBD), is the principal player in determining the host range of coronaviruses^11^. SARS-CoV-2 utilizes human ACE2 (hACE2) as an essential cellular receptor for infection^5,12^. Complex structural determinations have revealed the interactions between the SARS-CoV-2 RBD and hACE2^13–16^. Cryo-EM studies revealed that the SARS-CoV-2 spike trimer, like that of SARS-CoV, needs to have at least one RBD in an “up” conformation to bind hACE2^17–24^. Therefore, a spike trimer with all three “down” RBDs is in a receptor-binding inactive state, and the conformational change of at least one RBD from “down” to “up” switches the spike trimer to a receptor-binding active state^18,24^. The spike and RBD of RaTG13 and SARS-CoV-2 share 97.5% and 89.2% amino acid sequence identity, respectively. Similarly, PCoV_GX (GenBank: QIA48614.1) shares 92.3% and 86.7% amino acid sequence identity with the SARS-CoV-2 in the spike and RBD, respectively. In contrast, PCoV_GD (GenBank: QLR06867.1) and SARS-CoV-2 have greater amino acid sequence identity in the RBD (96.9%) than in the spike protein (89.6%). Consistently, the RBD of PCoV_GD has demonstrated stronger binding to hACE2 than the RBD of RaTG13, and hACE2 also supported more efficient cell entry of the PCoV_GD than the RaTG13 pseudoviruses^25^. Data have not been reported regarding the binding of PCoV_GX spike and its RBD with hACE2 or whether hACE2 supports PCoV_GX pseudovirus cell entry.

Here we report the cryo-EM structures of the RaTG13 and PCoV_GX spikes at 2.93 Å and 2.48 Å resolution, respectively. These two spikes have all three RBDs in the “down” conformation. The comparisons of the RaTG13, PCoV_GX, and SARS-CoV-2 spike structures, the strength of their binding with hACE2, and their efficiency in facilitating pseudovirus cell entry provide important insights into the evolution and cross-species transmission of SARS-CoV-2.

## Results

### Protein expression and structure determination

The cDNAs encoding the PCoV_GX (GenBank: QIA48614.1) and RaTG13 (GenBank: QHR63300.2) spikes were synthesized with codon optimization for recombinant expression. The PCoV_GX ectodomain (residues 1-1205) was cloned into the pCAG vector and the RaTG13 ectodomain (residues 1–1209) into the pFastBac-Dual vector. Both constructs include a C-terminal foldon tag for trimerization, a Strep tag for purification, and the ‘2 P’ mutation for protein stabilization (K980P and V981P for PCoV_GX; K982P and V983P for RaTG13). The PCoV_GX spike was expressed and purified from FreeStyle 293-F cells, and the RaTG13 spike from Hi5 insect cells. Both proteins existed as heavy glycosylated homotrimers with no cleavage into the S1 and S2 subunits by endogenous proteases (Supplementary Fig. 1). Cryo-EM images were recorded using an FEI Titan Krios microscope operating at 300 kV with a Gatan K3 Summit direct electron detector. For the PCoV_GX and RaTG13 spike trimers, ~700,000 and ~450,000 particles, respectively, were subjected to 2D classification, and a total of 263,842 and 99,241 particles were selected and subjected to 3D refinement with C3 symmetry to generate density maps (Supplementary Fig. 2). The overall density maps were solved to 2.48 Å for the PCoV_GX spike and 2.93 Å for the RaTG13 spike (gold-standard Fourier shell correlation = 0.143) (Supplementary Fig. 3). The high-resolution density maps enabled us to build nearly all residues of the PCoV_GX spike (residues 14–1138) with 93 N-linked glycans and the RaTG13 spike (residues 14–1133) with 54 N-linked glycans (Supplementary Fig. 4). Data collection and refinement statistics for these two structures are listed in Supplementary Table 1.

### Overall structures of RaTG13 and PCoV_GX spikes

**Fig. 1:**
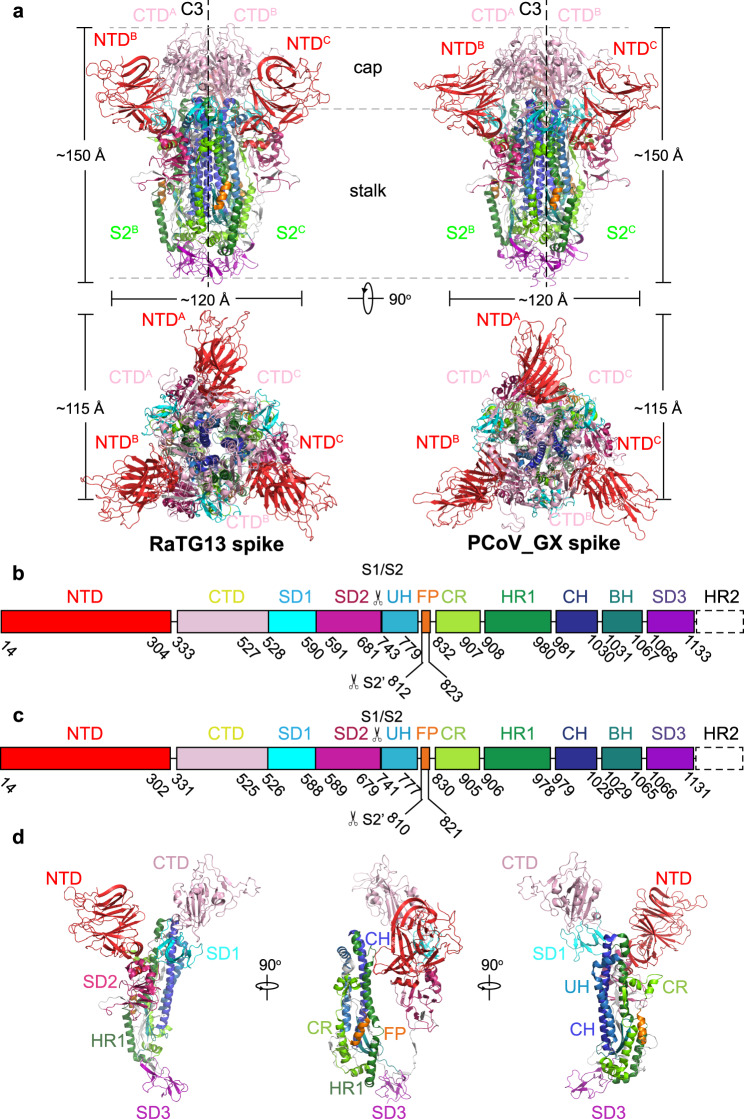
Overall structures of the RaTG13 and PCoV_GX spike glycoproteins. **a** Overall structures of the RaTG13 and PCoV_GX spike glycoproteins shown in side view (upper panel) and top view (lower panel). The domains were painted with colors shown in **b** and **c**. The trigonal axes are shown as black dashed lines. Visible segments of each monomer are labeled accordingly. The cap and stalk parts are partitioned by gray dashed lines. **b** Schematic representation of the RaTG13 spike monomer structural domains. The domains of RaTG13 are shown as boxes with the width related to the length of the amino acid sequence. The start and end amino acids of each segment are labeled. The position of the S1/S2 and S2’ cleavage sites are indicated by scissors. NTD, N-terminal domain; CTD, C-terminal domain; SD1, subdomain 1; SD2, subdomain 2; UH, upstream helix; FP, fusion peptide; CR, connecting region; HR1, heptad repeat 1; CH, central helix; BH, β-hairpin; SD3, subdomain 3. **c** Schematic representation of the PCoV_GX spike monomer structural domains. The abbreviations of elements are the same as in **b**. **d** Cartoon diagrams depicting three orientations of the spike monomer colored as in **b** and **c**. As the RaTG13 and PCoV_GX spike monomers have extremely similar structures, thus only the RaTG13 spike monomer was used to show the detailed architecture.

The overall structures of homotrimeric RaTG13 and PCoV_GX spikes resemble the previously reported pre-fusion structures of coronavirus spikes (Fig. 1a). Both spikes have a mushroom-like shape (~150 Å in height and ~120 Å in width), consisting of a cap mainly formed by β-strands and a stalk mainly formed by α-helices (Fig. 1a). Like other coronaviruses, the RaTG13 and PCoV_GX spike monomers are composed of the S1 and S2 subunits with a protease cleavage site between them (Figs. 1b, 1c). The structural components of the spike include the N-terminal domain (NTD), C-terminal domain (CTD), subdomain 1 (SD1) and subdomain 2 (SD2) in the S1 subunit; and the upstream helix (UH), fusion peptide (FP), connecting region (CR), heptad repeat 1 (HR1), central helix (CH), β-hairpin (BH), subdomain 3 (SD3) and heptad repeat 2 (HR2) in the S2 subunit (Fig. 1d, Supplementary Fig. 5). The rmsd for aligned Cα atoms is 0.708 Å between the RaTG13 and PCoV_GX spikes, indicating that they are very similar in the structure.

**Fig. 2:**
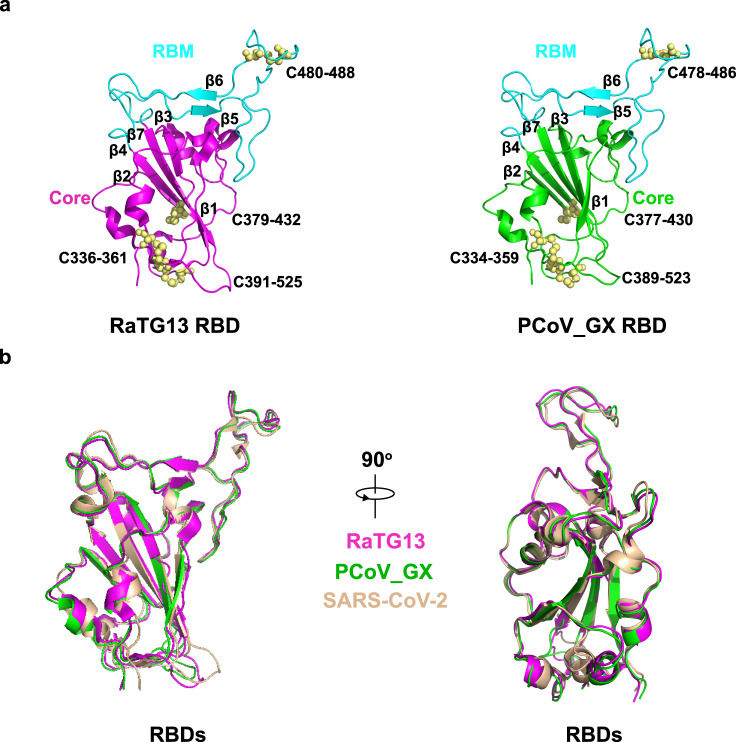
RBD structures of the RaTG13 and PCoV_GX spike proteins. **a** The RaTG13 and PCoV_GX RBDs are shown in side view. The RaTG13 RBD core is colored in magenta and the RBM in cyan; the PCoV_GX RBD core is colored in green and the RBM in cyan. Disulfide bonds are shown as yellow spheres with residues labeled. **b** Structural alignment of the RaTG13, PCoV_GX, and SARS-CoV-2 (PDB ID: 6M0J) RBDs. The RaTG13 RBD is colored in magenta, the PCoV_GX RBD is colored in green, and the SARS-CoV-2 RBD is in wheat. Aligned structures are shown in two orientations.

The RaTG13 and PCoV_GX spikes have the typical β-coronavirus structural features^26^. Their NTDs have a core consisting of three β-sheets plus one helix and a ceiling structure above the core (Supplementary Fig. 6). Three conserved disulfide bonds found in other β-coronavirus NTDs are also present in the NTDs of RaTG13 (15C-136C, 131C-166C, and 291C-301C) and PCoV_GX (15C-134C, 129C-164C, and 289C-299C) (Supplementary Fig. 6). Most β-coronaviruses utilize the CTD, also named receptor-binding domain (RBD), to specifically bind host receptor. The RaTG13 and PCoV_GX RBDs adopt a typical β-coronavirus RBD architecture, consisting of a β sheet core and an inserted loop called a receptor-binding motif (RBM) (Fig. 2a). The RaTG13 and PCoV_GX RBD cores are comprised of a twisted five^−^stranded antiparallel β sheet (β1, β2, β3, β4, and β7) with connecting loops and helices (Fig. 2a). The RBM, a long loop with two short β strands (β5 and β6), is inserted between the β4 and β7 strands (Fig. 2a). Besides three disulfide bonds in the core (336C-361C, 379C-432C, and 391C-525C in RaTG13; 334C-359C, 377C-430C, and 389C-523C in PCoV_GX) that stabilize the β sheet, the RaTG13 and PCoV_GX RBDs also have an additional disulfide bond (480C-488C in RaTG13 and 478C-486C in PCoV_GX) that connects the loop at the distal end of the RBM (Fig. 2a). The rmsd for aligned Cα atoms is 0.91 Å between the RaTG13 and SARS-CoV-2 RBDs and 0.59 Å between the PCoV_GX and SARS-CoV-2 RBDs. Therefore, the RaTG13, PCoV_GX, and SARS-CoV-2 RBDs are highly similar in the overall structure, even in the RBM for receptor binding (Fig. 2b).

### Conformations of the RaTG13 and PCoV_GX spikes

In our cryo-EM study, only conformational states of the RaTG13 and PCoV_GX spikes with all three “down” RBDs were captured. Structures of the SARS-CoV-2 spike with all three “down” RBDs were previously determined (PDB IDs: 6VXX and 6ZGE)^19,21^. In 6VXX, one of the SARS-CoV-2 RBDs exhibits contacts with 11 amino acid residues and the N234-linked glycans from the counter-clockwise monomer, and 5 amino acid residues from the clockwise monomer (Supplementary Fig. 7, Supplementary Table 2). In 6ZGE, the three “down” RBDs are more compactly packed, with one RBD having interactions with 35 residues and the N165/N234-linked glycans of the two neighboring monomers (Supplementary Fig. 7, Supplementary Table 2). In the RaTG13 spike, one RBD contacts 28 residues and the N165/N234/N370-linked glycans from the counter-clockwise monomer, and 13 residues from the clockwise monomer (Supplementary Fig. 7, Supplementary Table 2). A nearly identical packing of RBDs was also observed in a recently reported RaTG13 spike structure (PDB ID: 6ZGF)^21^. In the PCoV_GX spike, the number of RBD-contacting residues is 27 from the counter-clockwise monomer and 16 from the clockwise monomer. The N163/N232/N368-linked glycans from the counter-clockwise monomer are also involved in contact with the RBD (Supplementary Fig. 7, Supplementary Table 2). The rmsd values of aligned Cα atoms after superimposing the RaTG13 spike onto 6ZGE and 6VXX are 2.0 Å and 3.6 Å, respectively. For the PCoV_GX spike, the rmsd is 1.2 Å to 6ZGE and 3.1 Å to 6VXX. Therefore, the RaTG13 and PCoV_GX spikes are more structurally similar to the SARS-CoV-2 spike in the compact state (6ZGE) than in the loose state (6VXX), although both are closed with three “down” RBDs.

**Fig. 3:**
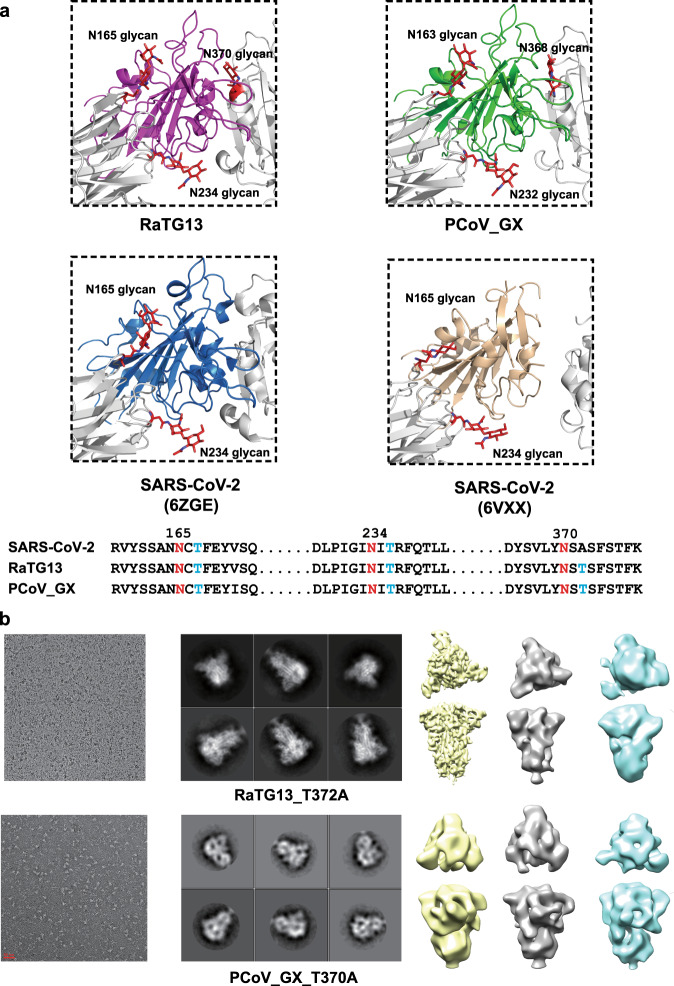
RBD-contacting glycans in the RaTG13 and PCoV_GX spikes. **a** Detailed structures of the RBD-glycans interface are shown. The RaTG13 RBD is colored in magenta, PCoV_GX RBD in green, SARS-CoV-2 (PDB ID: 6VXX) RBD in wheat, and SARS-CoV-2 (PDB ID: 6ZGE) RBD in marine; remaining regions shown in gray. Glycans are shown as red sticks and labeled. Sequence alignment of the SARS-CoV-2, RaTG13 and PCoV_GX RBD-contacting glycosylation sites is shown in the bottom panel. Sequences between the three sites are omitted and indicated by black dots. Amino acid positions of asparagines are indicated above the sequences according to SARS-CoV-2. Asparagines (N) are colored red and threonines (T) are colored blue. **b** Cryo-EM analysis of the RaTG13_T372A and negative-staining EM analysis of the PCoV_GX_T370A. The representative micrographs, 2D classification and 3D classification results were shown.

The capture of only compact and closed state suggested that the RaTG13 and PCoV_GX spikes are more prone to adopt the receptor-binding inactive state. The number of protein-protein interactions around the “down” RBD is nearly the same among the RaTG13, PCoV_GX and SARS-CoV-2 (6ZGE) spikes. In the RaTG13 and PCoV_GX spikes, the N165/N234/N370-linked glycans, spatially positioned at three vertices of a triangle, contact the RBD (Fig. 3a). The N370-linked glycans is not observed in the SARS-CoV-2 spike because the -NSA- motif is not a glycosylation site (Fig. 3a). Previous studies by Casalino et al.^27^ and Henderson R et al.^28^ showed that glycans at the N165 and N234 sites are involved in modulating the conformational dynamics of the SARS-CoV-2 RBD. All these results led us to investigate the role of the N370-linked glycans in the modulation. We generated the RaTG13_T372A and PCoV_GX_T370A mutants to delete this glycosylation site by mimicking the -NSA- motif in the SARS-CoV-2 spike. By 2D and 3D classifications of negative-staining or cryo-EM data, only the conformation with all three “down” RBDs was captured for these two mutants, indicating that deletion of the N370-linked glycans is not enough to efficiently switch the RBD from the “down” to “up” conformation in the RaTG13 and PCoV_GX spikes (Fig. 3b).

**Fig. 4:**
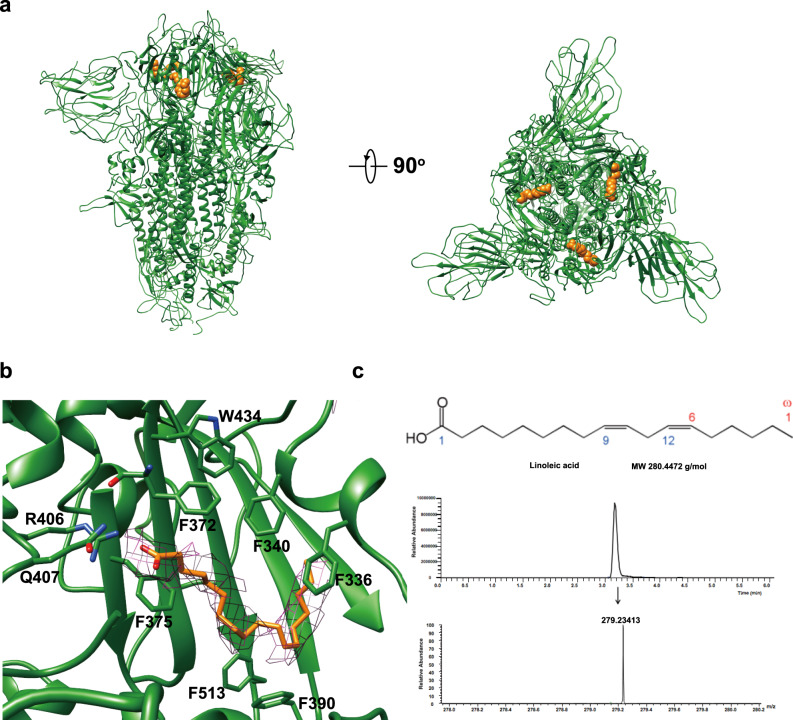
Binding of linoleic acid (LA) with the PCoV_GX spike. **a** LA-bound PCoV_GX spike shown in side view and top view. The LA is shown as orange spheres. **b** LA in the pocket of PCoV_GX spike RBD. LA is shown as orange sticks with EM density and PCoV_GX spike is colored green. LA-interacting amino acid residues are shown as sticks. **c** LC-MS analysis of extracted lipids from the purified PCoV_GX spike from two independent experiments. The chemical structure and molecular weight of LA are shown in the top panel, C18 column elution profile in the middle panel, and the molecular weight of corresponding peak in the bottom panel.

It has been recently revealed that the SARS-CoV-2 spike (PDB ID: 6ZB5) binds free fatty acid linoleic acid (LA) in a pocket in the RBD, which helps stabilize the spike in the compact and closed conformation^29^. We also found extra density in the nearly same pocket in the map of PCoV_GX spike (Fig. 4a, b). The LA could be well modeled into the density and liquid chromatography coupled mass spectrometry (LC-MS) confirmed the presence of LA in the purified PCoV_GX spike (Fig. 4c). Structural comparison showed a nearly identical binding mode of the LA by the PCoV_GX and SARS-CoV-2 spikes (Supplementary Fig. 8). In both spikes, the LA is located into a hydrophobic pocket mostly shaped by phenylalanine residues. An arginine (PCoV_GX R406 and SARS-CoV-2 R408) and a glutamine (PCoV_GX Q407 and SARS-CoV-2 Q409) provide the anchor for interacting the headgroup carboxyl of the LA (Supplementary Fig. 8). The phenylalanine, arginine, and glutamine residues around the LA-binding pocket are also conserved in the RaTG13 spike. However, no density for the bound-LA was observed in our map of the RaTG13 spike, as well as in the map of the RaTG13 spike reported by another group (PDB ID: 6ZGF) (Supplementary Fig. 8).

### Binding of hACE2 with the RBDs

**Fig. 5:**
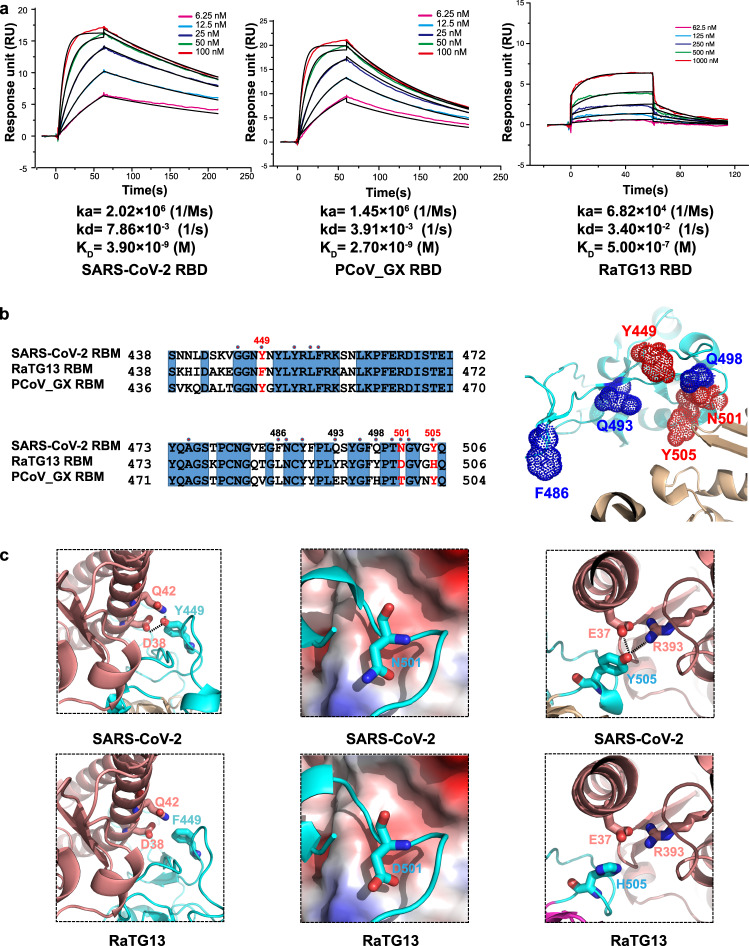
Binding affinities and interactions of human ACE2 with different RBDs. **a** Binding curves of immobilized human ACE2 with the SARS-CoV-2 (left panel), PCoV_GX (middle panel), or RaTG13 (right panel) RBD. Data are shown as different colored lines and the best fit of the data to a 1:1 binding model is shown in black. **b** Sequence alignment of the RBMs from the SARS-CoV-2, PCoV_GX, and RaTG13 spikes (left panel). Residues in the SARS-CoV-2 RBM that contact with hACE2 are indicated by red dots. The SARS-CoV-2 RBD (colored in cyan) and hACE2 (colored in wheat) are shown as cartoon (right panel, PDB ID: 6M0J). Six residues Y449, F486, Q493, Q498, N501, and Y505 in the RBM of SARS-CoV-2 that are not conserved in the RaTG13 are shown as dots. **c** Important sites accounting for different binding strength of hACE2 with the RaTG13 and SARS-CoV-2 RBDs (SARS-CoV-2 RBD-hACE2 interface: PDB ID 6M0J). The RBDs are showed as cyan cartoon and the hACE2 is showed in wheat cartoon or surface with electrostatic potential. Hydrogen bonds between SARS-CoV-2 Y449 and hACE2 D38 and Q42 would be abolished after Y to F mutation in the RaTG13 RBM (two leftmost panels). SARS-CoV-2 N501 mutation to D501 in RaTG13, contacting the hACE2 local negatively charged surface would disfavor the binding of hACE2 with the RaTG13 RBD (two middle panels). Hydrogen bonds between SARS-CoV-2 Y505 and hACE2 E37 and R393 would be abolished after Y to H mutation in the RaTG13 RBM (two rightmost panels).

We measured the binding affinities of hACE2 with the RBDs of RaTG13, PCoV_GX, and SARS-CoV-2 using surface plasmon resonance (SPR). The PCoV_GX and SARS-CoV-2 RBDs bound to hACE2 with comparable affinities of 2.7 nM and 3.9 nM, respectively. However, the RaTG13 RBD bound to hACE2 with a much weaker affinity of 500 nM (Fig. 5a). Sequence comparisons showed that both the RaTG13 and PCoV_GX RBMs share 75.3% amino acid sequence identity with the RBM of SARS-CoV-2. Of the 16 residues in the SARS-CoV-2 RBM involved with ACE2 binding, ten are conserved in the RaTG13, PCoV_GX, and SARS-CoV-2 (Fig. 5b). The other six SARS-CoV-2 residues that are not conserved in the RaTG13 and PCoV_GX are Y449, F486, Q493, Q498, N501, and Y505 (Fig. 5b). Except for F486, which is replaced by a leucine in the RaTG13 and PCoV_GX, Y449, Q493, Q498, N501, and Y505 in the SARS-CoV-2 RBM form a patch that has significant hydrophilic interactions with hACE2 (Fig. 5b). To experimentally investigate important sites for different binding strength of hACE2 with the RaTG13 and SARS-CoV-2 RBDs, we generated six single-site mutants of the RaTG13 RBD (F449Y, L486F, Y493Q, Y498Q, D501N, and H505Y) by changing residues at these sites to corresponding residues of the SARS-CoV-2 (Supplementary Fig. 9). The SPR experiments showed that the D501N mutation resulted in the most significant increase (~9 fold) of the binding strength with hACE2 (K_D_ = 57.4 nM) among all six mutations (Supplementary Fig. 10). The F449Y and H505Y mutations also modestly increased the binding with hACE2 by ~2- and ~3-fold, respectively (Supplementary Fig. 10). We also generated two RaTG13 RBD double-site mutants (F449Y/D501N and D501N/H505Y), which bound hACE2 more tightly than wild-type RBD with an affinity of 23.9 nM and 8.1 nM, respectively (Supplementary Fig. 9, 10). These results collectively support that 449, 501, and 505 are important sites accounting for different binding strength of hACE2 with the RaTG13 and SARS-CoV-2 RBDs.

The SARS-CoV-2 RBD-hACE2 complex structure revealed that at the 449 site, SARS-CoV-2 RBD has a tyrosine forming two hydrogen bonds with hACE2 D38 and Q42 upon binding. This tyrosine is conserved in the PCoV_GX RBD but is replaced by a phenylalanine in the RaTG13 RBD, which is expected to disrupt the hydrogen-bonding interactions (Fig. 5c, Supplementary Fig. 11). Similarly, SARS-CoV-2 Y505 forms two hydrogen bonds with hACE2 E37 and R393. This residue is conserved in the PCoV_GX RBD, whereas the histidine found at this site in the RaTG13 RBD may alter interactions with hACE2 (Fig. 5c, Supplementary Fig. 11). Regarding to the 501 position, both SARS-CoV-2 N501 and PCoV_GX T499 have the electrical neutral side-chain involved in hACE2 binding. Considering that hACE2 has a local negatively charged surface surrounding the 501 position, the change to acidic D501 is expected to disfavor the binding of hACE2 with the RaTG13 RBD, compared with the SARS-CoV-2 and PCoV_GX RBDs (Fig. 5c, Supplementary Fig. 11).

### Binding of hACE2 with the spikes and the entry efficiency of pseudoviruses

**Fig. 6:**
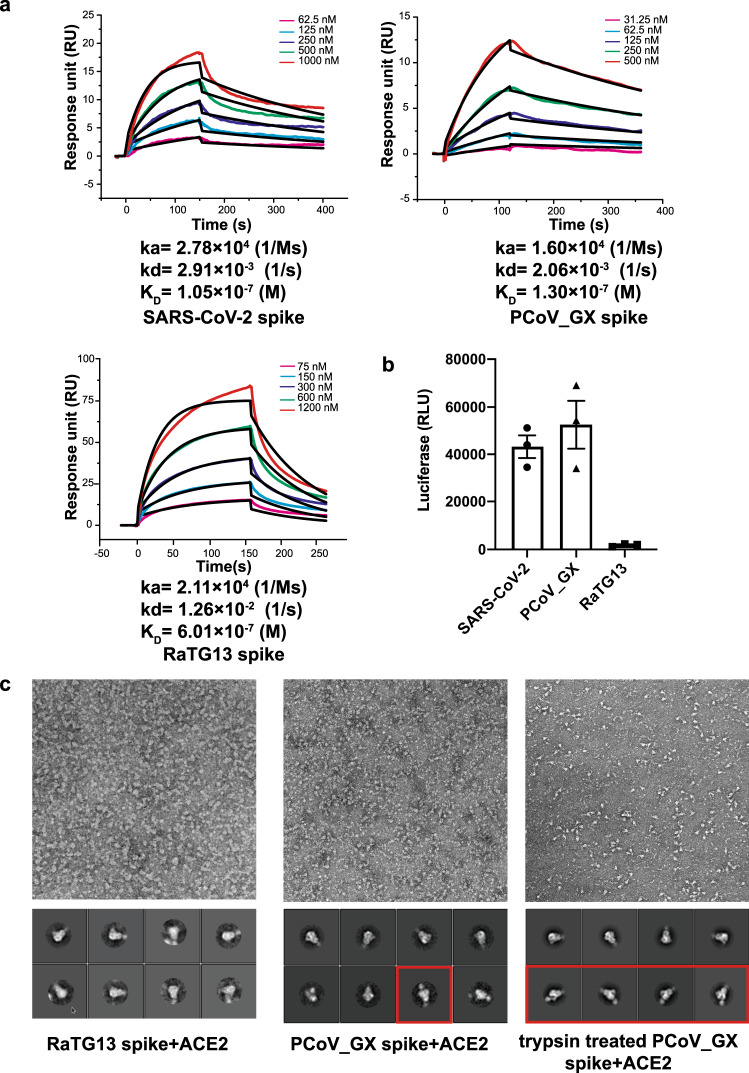
Binding affinities of human ACE2 with different spikes and the cell entry of pseudoviruses. **a** Binding curves of immobilized hACE2 with the SARS-CoV-2, PCoV_GX, or RaTG13 spike. Data are shown as different colored lines and the best fit of the data to a 1:1 binding model is shown in black. **b** The cell entry efficiencies of pseudoviruses as measured by luciferase activity. SARS-CoV-2, PCoV_GX, and RaTG13 pseudoviruses were used to infect hACE2-transfected HEK 293 T cells. Data shown are from three independent experiments. Data are presented as mean values ± SEM. **c** The representative micrographs and 2D classification results of negative-staining EM. Both spikes were incubated with 4-fold molar ratio of hACE2. The red boxes show the complex of the PCoV_GX spike with hACE2.

We also measured the binding affinities of hACE2 with the spikes of RaTG13, PCoV_GX, and SARS-CoV-2. Interestingly, we found that despite exhibiting the receptor-binding inactive conformation in the cryo-EM images, the PCoV_GX spike bound hACE2 with an affinity of 130 nM, comparable to the 105 nM affinity of the SARS-CoV-2 spike. The binding of RaTG13 spike to hACE2 was weaker, with an affinity of 601 nM (Fig. 6a). We tested the entry of the RaTG13, PCoV_GX, and SARS-CoV-2 pseudoviruses into HEK 293 T cells expressing hACE2. Consistently, the PCoV_GX and SARS-CoV-2 pseudoviruses had comparable entry efficiency, whereas the RaTG13 pseudovirus exhibited little to no entry (Fig. 6b).

To capture the hACE2-bound state of the RaTG13 and PCoV_GX spikes, we mixed spike and hACE2 at a 1:4 molar ratio and performed negative-staining EM. The 2D classification did not show particles with bound hACE2 for the RaTG13 spike, but ~9% of the PCoV_GX particles were bound to hACE2. After treating the PCoV_GX spike with trypsin for 2 h, the ratio of hACE2-bound particles increased to 20% (Fig. 6c). These results further support that the conformational switch of the spike is a dynamic equilibrium process and that binding of hACE2 would capture the spike with “up” RBDs and shift the process towards more spikes ready for receptor binding and membrane fusion.

## Discussion

Coronavirus spike glycoproteins recognize their host cellular receptor and mediate membrane fusion for entry, thereby functioning as the most critical coronavirus protein in determining viral evolution and cross-species transmission. Thus, comparative studies of the spikes from evolution-related coronaviruses have important implications for viral evolution and cross-species transmission. Coronaviruses RaTG13 isolated from bat and PCoV_GX isolated from pangolin are closely related to the SARS-CoV-2. Here we determined the high-resolution cryo-EM structures of the RaTG13 and SARS-CoV-2 spikes. Together with the SARS-CoV-2 spike, the comparisons of their structures, the strength of their binding with hACE2, and their efficiency in facilitating pseudovirus cell entry provide important insights into the evolution and cross-species transmission of the SARS-CoV-2.

Most β-coronaviruses utilize the RBD to specifically bind the host receptor. Compared to other structural components in the spike, the RBD harbors the most sequence and structure variations across different β-coronaviruses and thus has important implications for viral evolution and cross-species transmission^26^. Our structural determinations of the RaTG13 and PCoV_GX spikes showed that their RBDs are very similar to the SARS-CoV-2 RBD in the overall structure. However, the SPR experiments showed that only PCoV_GX RBD exhibited a hACE2-binding affinity comparable to that of the SARS-CoV-2 RBD, whereas the RaTG13 RBD demonstrated far weaker hACE2 binding. Sequence alignments indicated amino acid variations at six sites (SARS-CoV-2 Y449, F486, Q493, Q498, N501, and Y505) may be responsible for the differences in hACE2-binding among the RaTG13, PCoV_GX, and SARS-CoV-2 RBDs. The residues Y449, Q493, Q498, N501, and Y505 cluster together to form a patch on the SARS-CoV-2 RBD that has significant hydrophilic interactions with hACE2. Our RaTG13 RBD mutagenesis and SPR experiments further pinpointed three important sites (Y449, N501, and Y505) accounting for the weak binding we observed between hACE2 and the RaTG13 RBD. Notably, the RaTG13 D501N mutant resulted in the most significant hACE2 binding enhancement (~9 fold) among all six sites we tested. Our findings are supported by recent reports of adapted and remodeled SARS-CoV-2 strains utilized in mouse model studies. Gu et al. reported an adapted SARS-CoV-2 strain with increased infectivity in mice that has a N501Y mutation in the RBD^30^. Dinnon et al. remodeled the SARS-CoV-2 RBD at two sites (Q498Y and P499T) to facilitate efficient binding to mouse ACE2, producing a recombinant virus that can effectively utilize mouse ACE2 for entry^31^. We further propose that the patch containing Y449, Q493, Q498, N501, and Y505 plays a critical role in the evolution of the SARS-CoV-2 RBD, promoting especially tight binding with hACE2 and impacting the varying affinities observed between the RBD and ACE2 orthologs in wild and domestic animals^25,32^.

Cryo-EM studies have revealed that the RBD “down” to “up” conformational switch is a prerequisite for hACE2 receptor binding by SARS-CoV-2^24^. The molecular basis of the efficient conformational sampling of the SARS-CoV-2 RBD is still not well understood. Factors that may be involved include the furin-cleavage site, the RBD-contacting glycans, as well as the recently reported free fatty acid-binding pocket in the RBD. In our cryo-EM study, we only captured the closed state of the RaTG13 and PCoV_GX spikes with all three RBDs adopting the “down” conformation. Therefore, in contrast to the SARS-CoV-2 spike which samples the receptor-binding inactive and active states efficiently^19–24^, the RaTG13 and PCoV_GX spikes are more prone to adopt the receptor-binding inactive state. Another group had the same conclusion in a recent structural determination of the RaTG13 spike^21^.

The roles of RBD-contacting glycans in the modulation of the SARS-CoV-2 spike state has been recently studied. With simulation and binding data, Casalino et al. suggested that that the N165 and N234-linked glycans are critical in stabilizing the “up” conformation of the RBD^27^. Hednerson et al. also revealed that deletion of the N234-linked glycans shift the RBD dynamics more toward the “down” state, whereas deletion of N165-linked glycans appeared to slightly enhance the distribution toward more “up” state using cryo-EM structure classification method^28^. Although the conclusions regarding the N165 site are different, both studies showed that the N165 and N234-linked glycans serve as control elements of the SARS-CoV-2 RBD conformational dynamics. Besides the N165 and N234 glycosylation sites, we observed an additional N370-linked glycans contacting the RBD in the RaTG13 and PCoV_GX spikes. This led us to wonder if the presence of the N370-glycans contributes to the closed state of the RaTG13 and PCoV_GX spikes and if the absence of it contributes to the more flexible SARS-CoV-2 RBDs. We investigated the RaTG13 and PCoV_GX spike mutants by EM classification, and the results showed that deletion of the N370-linked glycans did not result in an “up” RBD. Although our EM results indicated that the N370 site may not play critical roles, the specific effects of the N165, N234, and N370 sites in the conformational modulation of the RaTG13 and PCoV_GX spike need to be further studied by extra methods, such as the more sensitive molecular simulation.

Besides the RBD-contacting glycans, the roles of essential free fatty acid in modulating the SARS-CoV-2 spike state has also been revealed recently^29^. The binding of the linoleic acid (LA) by the RBD into a pocket stabilizes the SARS-CoV-2 spike into a locked and closed state, giving rise to reduced ACE2 interaction. Interestingly, the PCoV_GX spike also has the specific LA binding into the nearly same pocket in the RBD. Although the pocket and interacting RBD residues are conserved, the LA did not bind to the RaTG13 spike purified from insect cells in our study, as well as to the RaTG13 spike purified from HEK293 cells in another study. It is still unknown why the LA selects the PCoV_GX and SARS-CoV-2 spikes, and whether the specific binding represents one evolutional event of the spike would be an interesting question to be further studied in the future.

The RaTG13 and PCoV_GX spikes and their RBDs all bound hACE2 in our SPR experiments, although both the RBD and spike of PCoV_GX exhibited higher binding affinities than those of RaTG13. These results suggest that the RaTG13 and PCoV_GX spikes can also spontaneously sample “up” RBD, which is essential for hACE2 binding. The reason for not observing these conformations in our cryo-EM study may be due to the ratio of the RaTG13 and PCoV_GX spike particles adopting this state being too low. Interestingly, the PCoV_GX spike bound to hACE2 with an affinity comparable to that of the SARS-CoV-2 spike and also had similar efficiency in cell entry. In contrast, the RaTG13 spike was much weaker in binding hACE2 and mediating cell entry. We also confirmed the binding of PCoV_GX spike to hACE2 by negative-staining EM.

In summary, the comparisons of the RaTG13, PCoV_GX, and SARS-CoV-2 spike structures, the strength of their binding with hACE2, and their efficiency in facilitating pseudovirus cell entry strongly suggest that tight RBD-hACE2 binding and efficient RBD “down” to “up” conformational change are both required for SARS-CoV-2 to gain highly efficient transmission capability.

## Methods

### Construct design

The cDNAs encoding the SARS-CoV-2 spike (GenBank: YP_009724390.1), PCoV_GX spike (GenBank: QIA48614.1), and RaTG13 spike (GenBank: QHR63300.2) were synthesized with codons optimized for protein expression (Supplementary Table 3). The SARS-CoV-2 spike ectodomain (1-1208) and PCoV_GX ectodomain (1-1205) were cloned into the pCAG vector separately, and the RaTG13 spike ectodomain (1-1209) was cloned into the pFastBac-Dual vector (Invitrogen). All the spike constructs included a C-terminal foldon tag for trimerization, a Strep tag for purification, and ‘2 P’ mutations (K986P and V987P for SARS-CoV-2, K980P, and V981P for PCoV_GX, K982P, and V983P for RaTG13). RaTG13_T372A and PCoV_GX_T370A spike mutants were generated by site-specific PCR mutagenesis and were cloned into the pCAG vector as described above (Supplementary Table 4).

The human ACE2 extracellular domain (19-615), SARS-CoV-2 RBD (333-527), PCoV_GX RBD (331-524), and RaTG13 RBD (333-526) were inserted into the pFastBac-Dual vector, with an N-terminal gp67 signal peptide for secretion and a C-terminal 6×His tag for purification. RaTG13 RBD mutants (F449Y, L486F, Y493Q, Y498Q, D501N, H505Y, F449Y/H505Y, and D501N/H505Y) were generated by site-specific PCR mutagenesis and cloned into the pCAG vector with an N-terminal CD4 signal peptide for secretion and a C-terminal 6×His tag for purification (Supplementary Table 4).

### Protein expression and purification

The SARS-CoV-2, PCoV_GX, RaTG13_T372A, and PCoV_GX_T370A spikes and RaTG13 RBD mutants were expressed in FreeStyle 293-F cells (Invitrogen) cultured in suspension in 5% CO_2_ atmosphere, at 37 °C with shaking at 120 rpm. Cell cultures were transiently transfected with 1 mg of plasmid per liter of culture at a density of 2 × 10^6^/ml using polyethylenimine (PEI) (Sigma), and the supernatants were collected 72 h later. RaTG13 spike, three RBDs (RaTG13, PCoV_GX, and SARS-CoV-2), and hACE2 were produced in Hi5 insect cells using the Bac-to-Bac baculovirus system (Invitrogen). Briefly, the construct was transformed into DH10Bac competent cells and the extracted bacmid was transfected into Sf9 cells using Cellfectin II reagent (Invitrogen). The baculoviruses were harvested after 7–9 days. The high-titer viruses were generated after one more amplification that used to infect Hi5 cells at a density of 2 × 10^6^/ml, and the supernatants were harvested after 60 h.

The supernatants containing the SARS-CoV-2, PCoV_GX, and RaTG13 spikes were concentrated and exchanged to binding buffer (50 mM Tris, pH 8.0, 150 mM NaCl), purified by StrepTactin beads (IBA), and then purified by gel-filtration chromatography using a Superose 6 column (GE Healthcare) pre-equilibrated with buffer containing 20 mM Tris pH 8.0 and 150 mM NaCl. The cell medium containing SARS-CoV-2 RBD, PCoV_GX RBD, hACE2, RaTG13 RBD and mutants were concentrated and exchanged to HBS buffer (10 mM HEPES, pH 7.2, 150 mM NaCl), purified by Ni-NTA resin (GE Healthcare), and followed by size-exclusion chromatography using a Superdex 200 column (GE Healthcare) pre-equilibrated with HBS buffer (10 mM HEPES, pH 7.2, 150 mM NaCl).

### Mass spectrometry analysis

To extract the fatty acids, 100 μl of the PCoV_GX protein sample was mixed with 400 μl organic reagents (dichloromethane: methonal = 2:1) on a shaker for a while, after stratification, centrifugation for 15 min at 3000 rpm. The organic phase was transferred to a glass vial and evaporated for 15 min by nitrogen. Afterward, 120 μl of solution (dichloromethane: methonal = 1:2) was added into the glass vial to dissolve the fatty acids. Subsequently, a Q-Exactive HF orbitrap mass spectrometer (Thermo Fisher, CA) with electrospray ionization (ESI) was used. The samples of 1 μl were passed over a Waters Cortecs C18 column (100*2.1 mm, 2.7 μm) heated to 40 °C using a gradient of 15% A to 98% B in 9 min (solvent A: 60% ACN + 40% H_2_O + 10 mM NH_4_Ac; solvent B: 90% IPA + 10% ACN). The source parameters are as follows: spray voltage: 3000 V; capillary temperature: 320 °C; heater temperature: 300 °C; sheath gas flow rate: 35 Arb; auxiliary gas flow rate: 10 Arb. Data analysis was performed by the software Xcalibur 3.0 (Thermo Fisher, CA).

### Surface plasmon resonance experiments

Running buffer composed of 10 mM HEPES pH 7.2, 150 mM NaCl and 0.05% (v/v) Tween-20 was used during the analysis, and all proteins were exchanged to the same buffer. The purified hACE2 was covalently immobilized to a CM5 sensor chip (GE Healthcare) via amine groups in 10 mM sodium acetate buffer (pH 4.5) to a level of around 700 response units using Biacore T200 (GE Healthcare). The blank channel of the chip was used as the negative control. Serial dilutions of the SARS-CoV-2, PCoV_GX, and RaTG13 spikes, their respective wild-type RBDs, and RaTG13 RBD mutants were flowed through the chip sequentially. The resulting data were analyzed using Biacore T200 Evaluation Software 3.1 (GE Healthcare) by fitting to a 1:1 binding model.

### Pseudovirus entry assays

SARS-CoV-2, PCoV_GX, and RaTG13 pseudoviruses were generated by co-transfection of human immunodeficiency virus backbones expressing firefly luciferase (pNL43R-E-luciferase) and pcDNA3.1 (Invitrogen) expression vectors encoding the respective spike protein into HEK 293 T cells (ATCC). Viral supernatants were collected 48–72 h later. The concentration of the harvested pseudovirus was normalized by a p24 ELISA kit (Beijing Quantobio Biotechnology Co., Ltd., China) before infecting hACE2-transfected 293 T cells. The infected cells were lysed 24 h after infection and viral entry efficiency was measured as luciferase activity by a Fire-Lucy assay kit (Vigorous Biotechnology Beijing Co., Ltd) using GraphPad Prism 8 for analysis.

### Trypsin treatment of the PCoV_GX and RaTG13 spike glycoproteins

L-(tosylamido-2-phenyl) ethyl chloromethyl ketone (TPCK)-treated trypsin was added to the purified PCoV_GX spike at a mass ratio of 1:100 in HBS buffer and incubated at room temperature for 2 h. SDS-PAGE was performed to determine that the spikes were fully cleaved into S1 and S2 fragments. The digestion reaction was stopped by applying the mixture to negative-staining.

### Negative-staining EM

The RaTG13, PCoV_GX, and trypsin-cleaved PCoV_GX spikes were separately mixed with hACE2 on ice for a few minutes at a molar ratio of 1:4. Aliquots of the spike-ACE2 mixture and the PCoV_GX_T372A spike at 0.05 mg/ml were deposited onto glow-discharged grids with a continuous carbon layer (Beijing Zhongjingkeyi Technology Co., Ltd.). The excess sample was removed using filter paper after 1 min of incubation on the grid, then washed twice, incubated with 5 μl of 2% uranyl acetate (UA) solution for another minute, then blotted with filter paper and air-dried at room temperature. These grids were examined under an FEI Tecnai Spirit electron microscope equipped with an FEI Eagle 4k CCD camera. Images were manually collected at a nominal magnification of 52,000× and at a defocus range between −1.5 and −1.8 μm, corresponding to a pixel size of 2.07 Å. Appropriately, 50–100 images were collected for each sample. Image format converting was conducted by EMAN2^33^. Particle auto-picking, particle extraction, and 2D classification were performed in RELION-3.1^34^.

### Cryo-EM sample preparation and data collection

Aliquots of spike ectodomains (4 μl, 0.3 mg/ml, in buffer containing 20 mM Tris pH 8.0 and 150 mM NaCl) were applied to glow-discharged holey carbon grids (Quantifoil grid, Au 300 mesh, R1.2/1.3) and grids with a layer of continuous ultrathin carbon film (Lacey carbon grid). The grids were then blotted and plunge-frozen into liquid ethane using Vitrobot Mark IV (Thermo Fisher Scientific).

Images for RaTG13_T370A spike were recorded using FEI Talos Arctica microscope (Thermo Fisher Scientific) operating at 200 kV and equipped with a Gatan K2 Summit direct electron detector (Gatan Inc.). Appropriate 200 movies were collected at a nominal magnification of 36,000× and at a defocus range between −1.5 and −2 μm with a pixel size of 1.17 Å. Each movie has a total accumulate exposure of 50 e^−^/Å^2^ fractionated in 32 frames.

Images for RaTG13 and PCoV_GX spike ectodomains were recorded using FEI Titan Krios microscope (Thermo Fisher Scientific) operating at 300 kV with a Gatan K3 Summit direct electron detector (Gatan Inc.) at Tsinghua University. The automated software (AutoEMation2^35^) was used to collect 3963 movies for PCoV_GX and 1889 movies for RaTG13 in super-resolution mode at a nominal magnification of 81,000× and at a defocus range between −1.5 and −1.8 μm. Each movie has a total accumulate exposure of 50 e^−^/Å^2^ fractionated in 32 frames of 175 ms exposure. The final image was binned 2-fold to a pixel size of 1.0825 Å. Data collection statistics are summarized in Supplementary Table 1.

### Cryo-EM data processing

Motion Correction (MotionCor2 v.1.2.6^36^), CTF-estimation (GCTF v.1.18^37^) and non-templated particle picking (Gautomatch v.0.56, http://www.mrc-lmb.cam.ac.uk/kzhang/) were automatically executed by TsingTitan.py program. Sequential data processing was carried out on RELION-3.1^34^. Initially, ~700,000 particles for PCoV_GX and ~450,000 particles for RaTG13 were subjected to 2D classification. After two or three additional 2D classification, the best selected 474,499 particles for PCoV_GX and 107,274 particles for RaTG13 were applied for the initial model and 3D classification.

For PCoV_GX, the best class (397,362 particles) from 3D classification yielded a resolution of 3.14 Å (with C3 symmetry). To improve map density, especially NTD and glycosides, particles were expanded with C3 symmetry, and then subjected to local search classification. The particles of best class from local search classification were further applied to CTF refinement with C3 symmetry and Bayesian polishing, which improved the resolution to 2.71 Å and 2.48 Å, respectively. Meanwhile, the selected particles were subjected to focused classification with an adapted mask on NTD, and then further applied to 3D-refinement, CTF refinement, and Bayesian polishing to reach a resolution of 3.64 Å. Additional 3D classification and Bayesian polishing resulted in the NTD map at a resolution of 3.68 Å with better quality. Three copies of NTD maps were fitted onto the whole structure map using Chimera v.1.15^38^, then combined together using PHENIX v.1.18.2^39^ combine_focused_maps.

For RaTG13, the best class (99,241 particles) from 3D classification were subjected to 3D auto-refine with C3 symmetry to generate a density map with a resolution of 2.93 Å. The reported resolutions were estimated with a gold-standard Fourier shell correlation (FSC) cutoff of 0.143 criterion. Local resolution variations were estimated using ResMap1.1.4^40^. Data processing statistics are summarized in Supplementary Table 1.

### Model building and refinement

The initial model of PCoV_GX and RaTG13 spikes were generated using the SWISS-MODEL^41^ and fit into the map using UCSF Chimera v.1.15^38^. Manual model rebuilding was carried out using Coot v.0.9.2^42^ and refined with PHENIX v.1.18.2^39^ real-space refinement. The quality of the final model was analyzed with Molprobity^43^ and EMRinger^44^ in PHENIX v.1.18.2^39^ and the glycan validation used Privateer^45^. The validation statistics of the structural models are summarized in Supplementary Table 1. All structural figures were generated using PyMOL 2.0^46^ and Chimera v.1.15^38^.

### Reporting summary

Further information on experimental design is available in the Nature Research Reporting Summary linked to this paper.

## Supplementary information

Supplementary Information

Reporting Summary

## Data Availability

The atomic coordinates of PCoV_GX spike and RaTG13 spike have been deposited in the Worldwide Protein Data Bank with the accession codes 7CN8 and 7CN4, respectively; the corresponding maps have been deposited in the Electron Microscopy Data Bank with the accession codes EMD-30418 and EMD-30416, respectively. Other structures for analysis including 6M0J, 6VXX, 6ZGE, 6ZGF, and 6ZB5 were obtained from the Protein Data Bank (PDB). Any other raw data pertaining to this study are available from the corresponding author upon reasonable request. Source data are provided with this paper.

## Source data

Source Data
