# Supplementary material for: Bat and pangolin coronavirus spike glycoprotein structures provide insights into SARS-CoV-2 evolution
Source: Nat Commun. 2021 Mar 11;12:1607. doi: 10.1038/s41467-021-21767-3 (PMC7952905; doi:10.1038/s41467-021-21767-3)
Supplement: Supplementary file 1 — Supplementary Information [file 41467_2021_21767_MOESM1_ESM.pdf]

Bat and pangolin coronavirus spike glycoprotein structures provide insights into SARS-CoV-2 evolution

Shuyuan Zhang[1,*], Shuyuan Qiao[1,*], Jinfang Yu[1,*], Jianwei Zeng[1,*], Sisi Shan[2], Long Tian[1], Jun Lan[1], Linqi Zhang[2], Xinquan Wang[1,#]

[1]The Ministry of Education Key Laboratory of Protein Science, Beijing Advanced Innovation Center for Structural Biology, Beijing Frontier Research Center for Biological Structure, Collaborative Innovation Center for Biotherapy, School of Life Sciences, Tsinghua University, 100084 Beijing, China
[2]Center for Global Health and Infectious Diseases, Comprehensive AIDS Research Center, Beijing Advanced Innovation Center for Structural Biology, School of Medicine, Tsinghua University, Beijing, China

*These authors contributed equally to this work
[#]Correspondence: xinquanwang@mail.tsinghua.edu.cn (X.W.)

Supplementary information
Supplementary Figures 1-11
Supplementary Tables 1-4

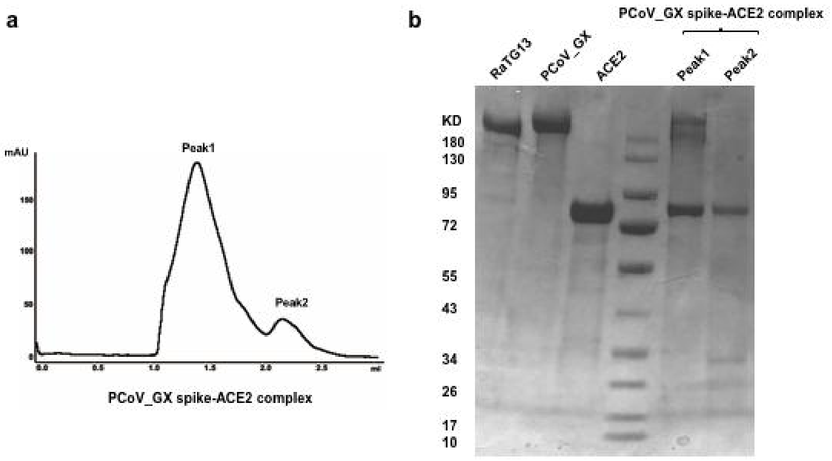

Supplementary Fig. 1 Purification of the RaTG13 spike, PCoV_GX spike and the PCoV_GX spike-hACE2 complex. (**a**) Size-exclusion chromatography elution profile of the PCoV_GX spike-hACE2 complex. Purified PCoV_GX spike was mixed with hACE2 at a molar ratio of 1:4 before applying to gel-filtration. (**b**) SDS–PAGE analysis of the gel-filtration elutions. Bands on the left side of marker are purified proteins (RaTG13 spike, PCoV_GX spike and hACE2) and bands on the right side are complex. Peak 1, PCoV_GX spike-hACE2 complex; Peak 2, excessive hACE2.

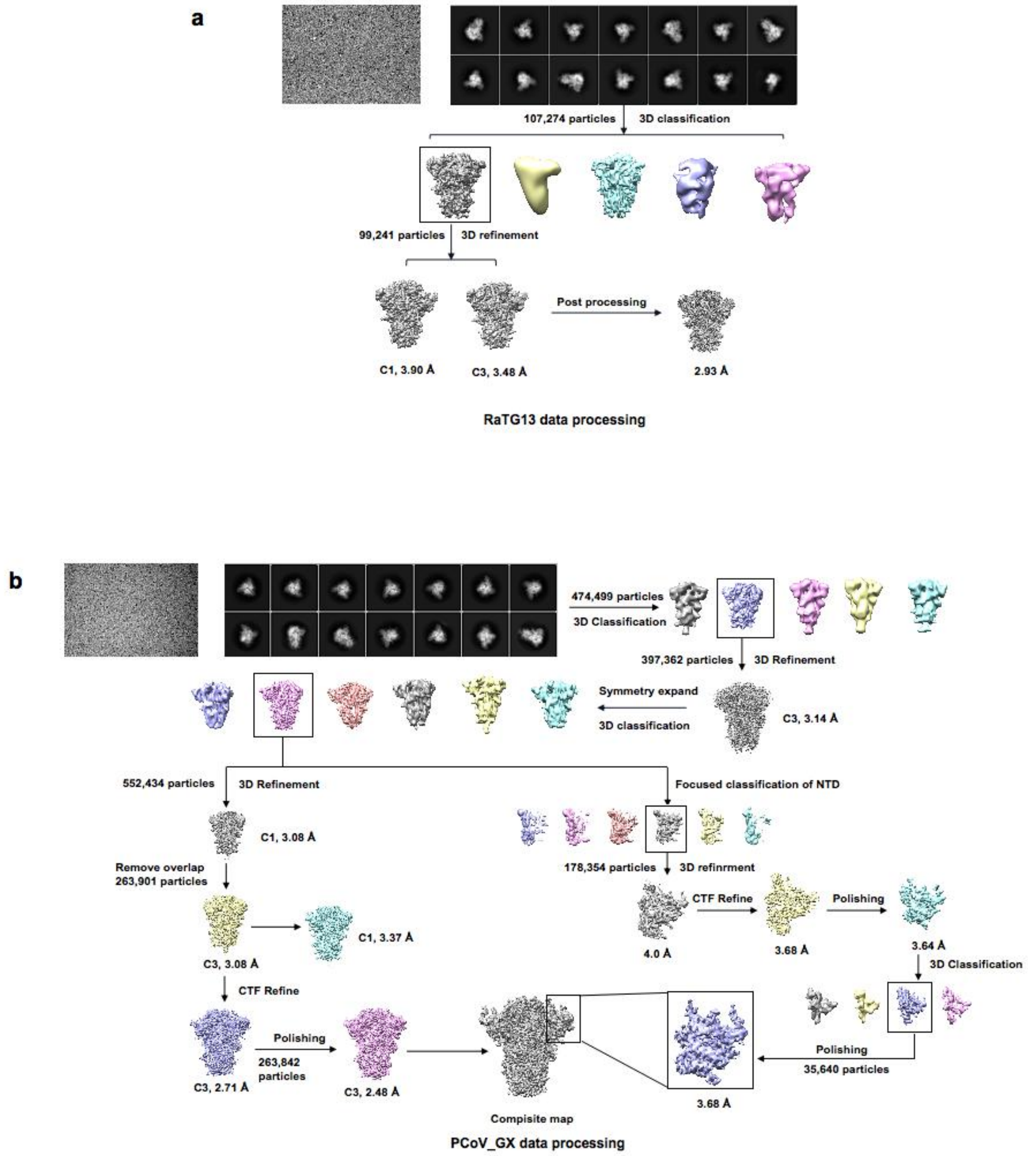

Supplementary Fig. 2 Cryo-EM data processing workflow. (**a**) Processing workflow of the RaTG13 spike cryo-EM data. (**b**) Processing workflow of the PCoV_GX spike cryo-EM data.

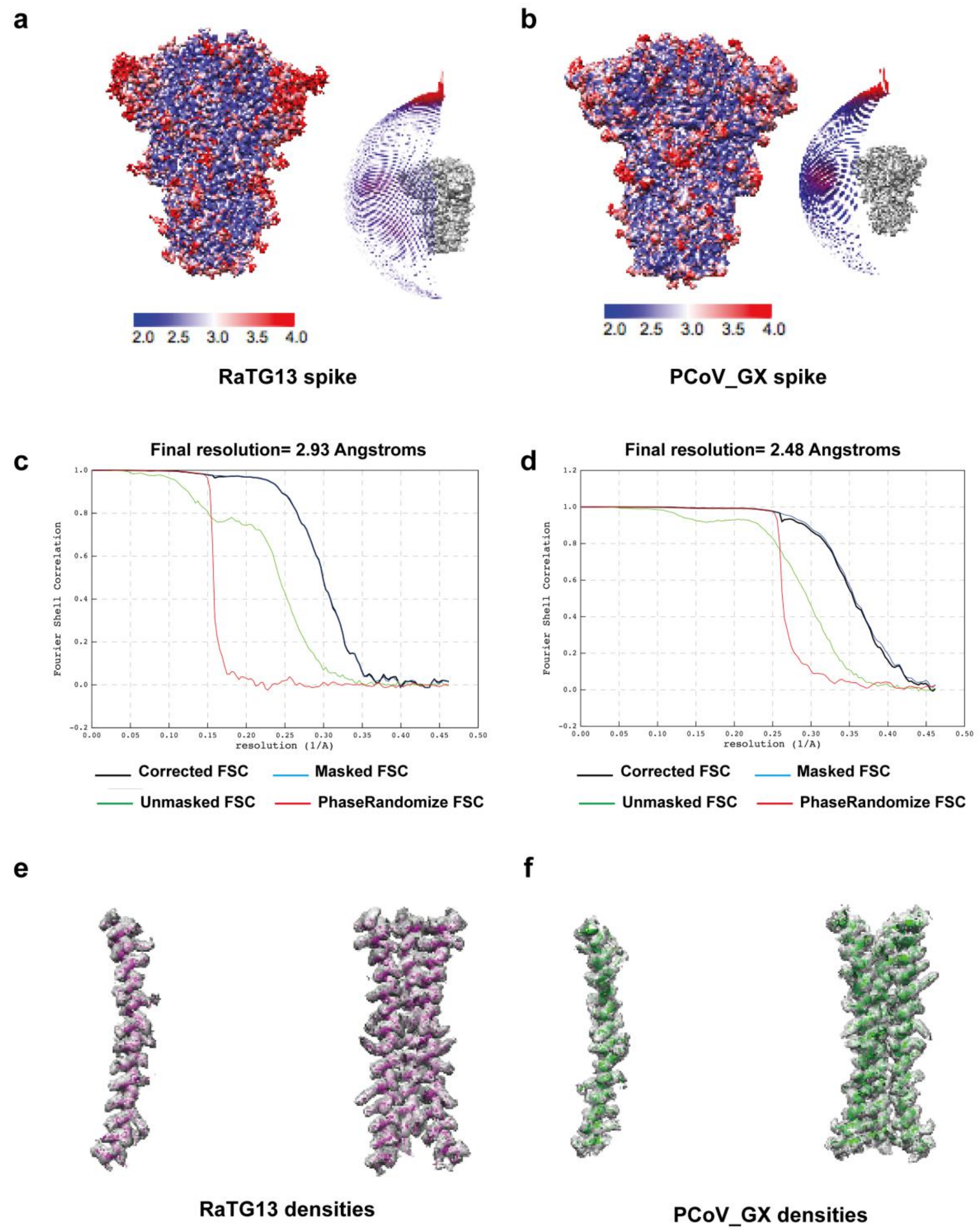

Supplementary Fig. 3 Cryo-EM structure validations. (**a**) (**b**) Local resolution map (left panel) and particle orientation distribution (right panel) of the RaTG13 spike (a) and the PCoV_GX spike (b). A color scale at the bottom of each local resolution map indicates resolution (2-4 Å). The angular distribution of the particles in each right panel is represented in the three-fold symmetric spike map. (**c**) (**d**) The corrected, unmasked, masked and phase randomised FSC for the cryo-EM reconstructions of the density maps of the RaTG13 spike and PCoV_GX spike with C3 symmetry. The final resolution of the RaTG13 spike is 2.93 Å. The final resolution of the PCoV_GX spike is 2.48 Å. (**e**) (**f**) Densities from the S2 regions (central helix) of the C3-refined RaTG13 spike (e) and PCoV_GX spike (f) structures. The map is contoured at 2.5 RMS to show the density.

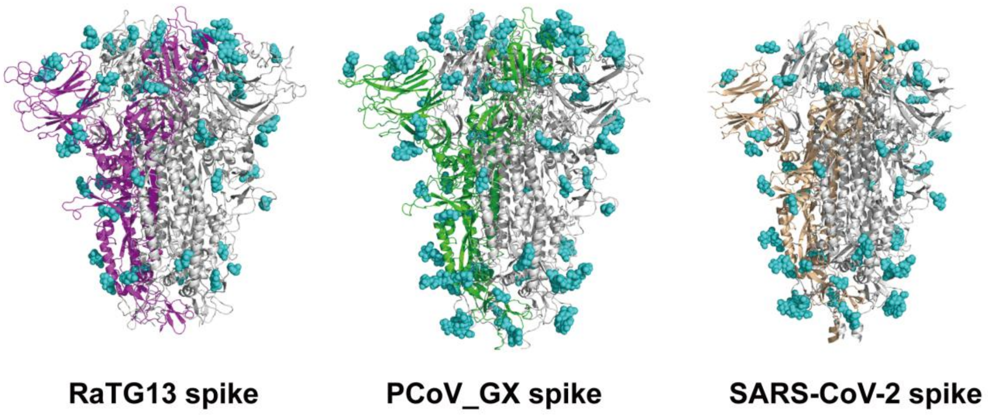

Supplementary Fig. 4 N-linked glycans of the RaTG13, PCoV_GX and SARS-CoV-2 spikes. A monomer for each spike trimer is colored magenta for RaTG13 in the left panel, green for PCoV_GX in the middle panel, and wheat for SARS-CoV-2 in the right panel (PDB ID: 6VXX). The remaining monomers for each spike trimer are shown in gray. Glycans for each spike are shown as cyan spheres.

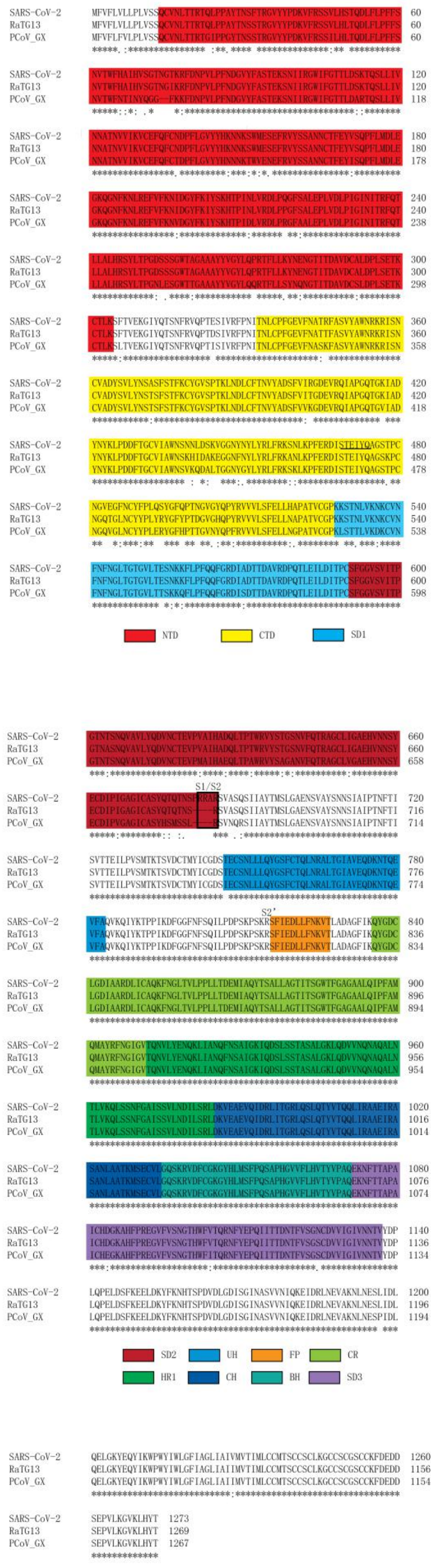
SARS-CoV-2
RaTG13
PCoV_GX
S1/S2
S2'
NTD
CTD
SD1
SD2
UH
FP
CR
HR1
CH
BH
SD3

Supplementary Fig. 5 Amino acid sequence alignment of the SARS-CoV-2, RaTG13 and PCoV_GX spikes. Identical residues are denoted by an "*" in the bottom consensus sequence row.

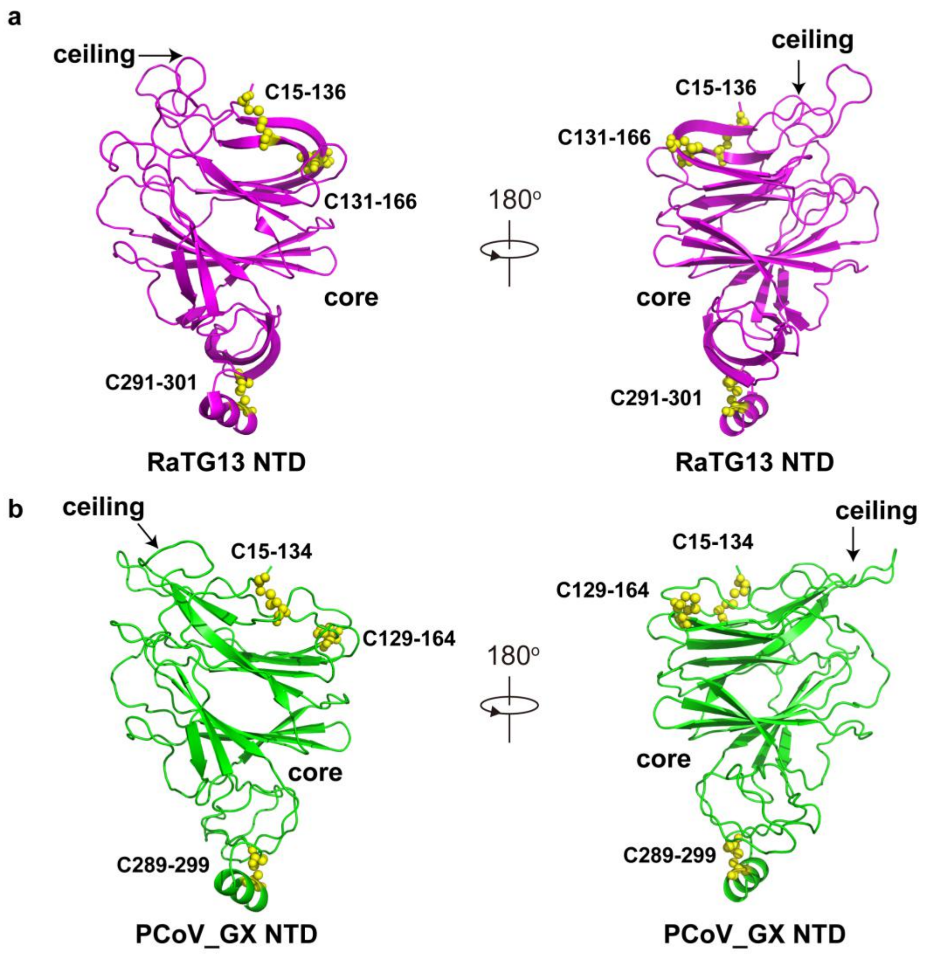

Supplementary Fig. 6 NTD structures of the RaTG13 and PCoV_GX spikes. (**a**) The RaTG13 NTD is shown in two opposite orientations and its NTD is colored magenta. (**b**) PCoV_GX NTD is shown in two orientations directions and its NTD is colored green. The ceiling region of the NTDs are indicated by black arrows, and the core regions are labeled. Disulfide bonds are shown as yellow spheres.

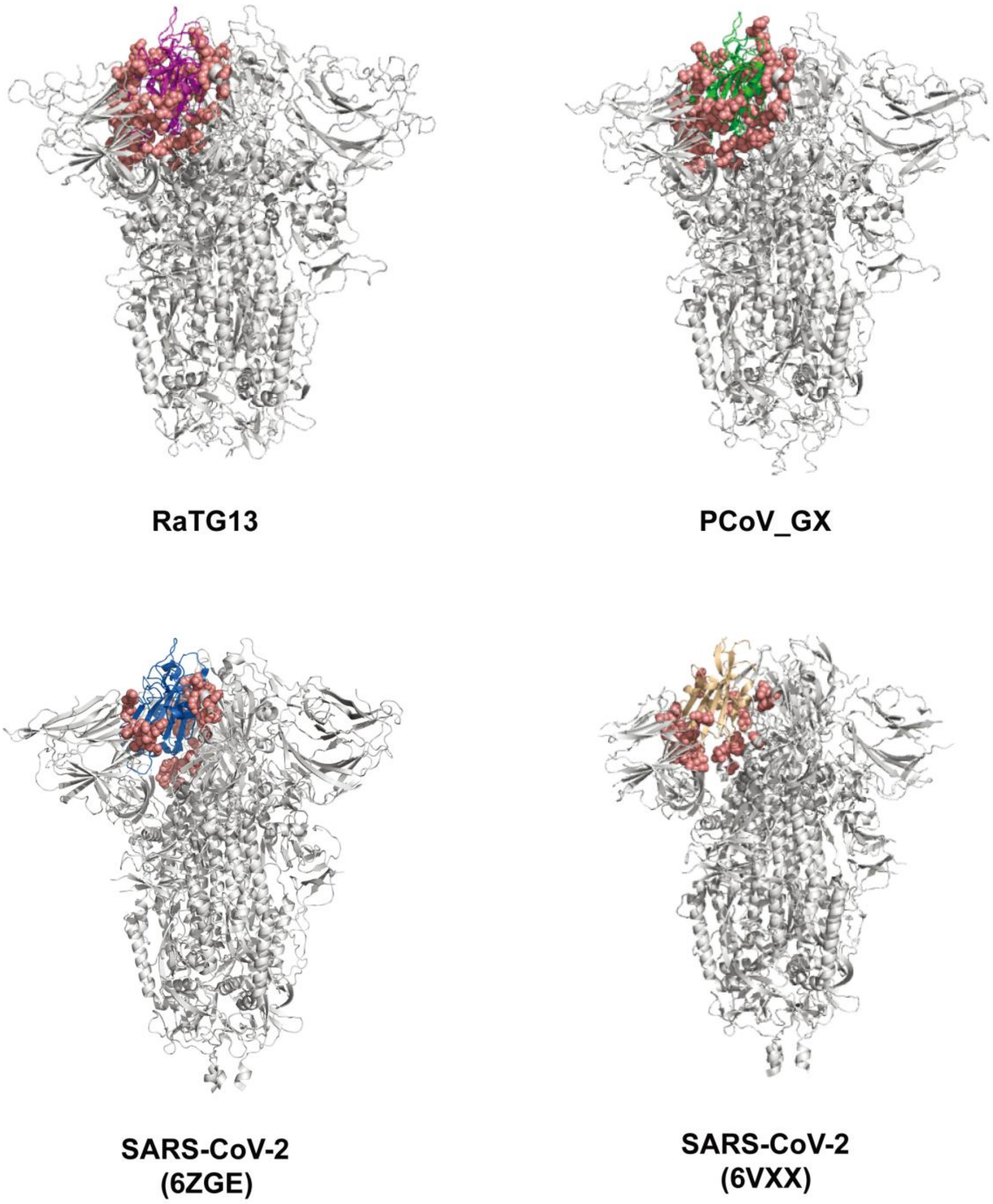

Supplementary Fig. 7 The amino acid residues and N-linked glycans interacting with one RBD. The RaTG13 RBD is colored in magenta, PCoV_GX RBD in green, SARS-CoV-2 (PDB ID: 6VXX) RBD in wheat, and SARS-CoV-2 (PDB ID: 6ZGE) RBD in marine; remaining regions shown in gray. The amino acid residues and glycans interacting with one RBD are shown as salmon spheres.

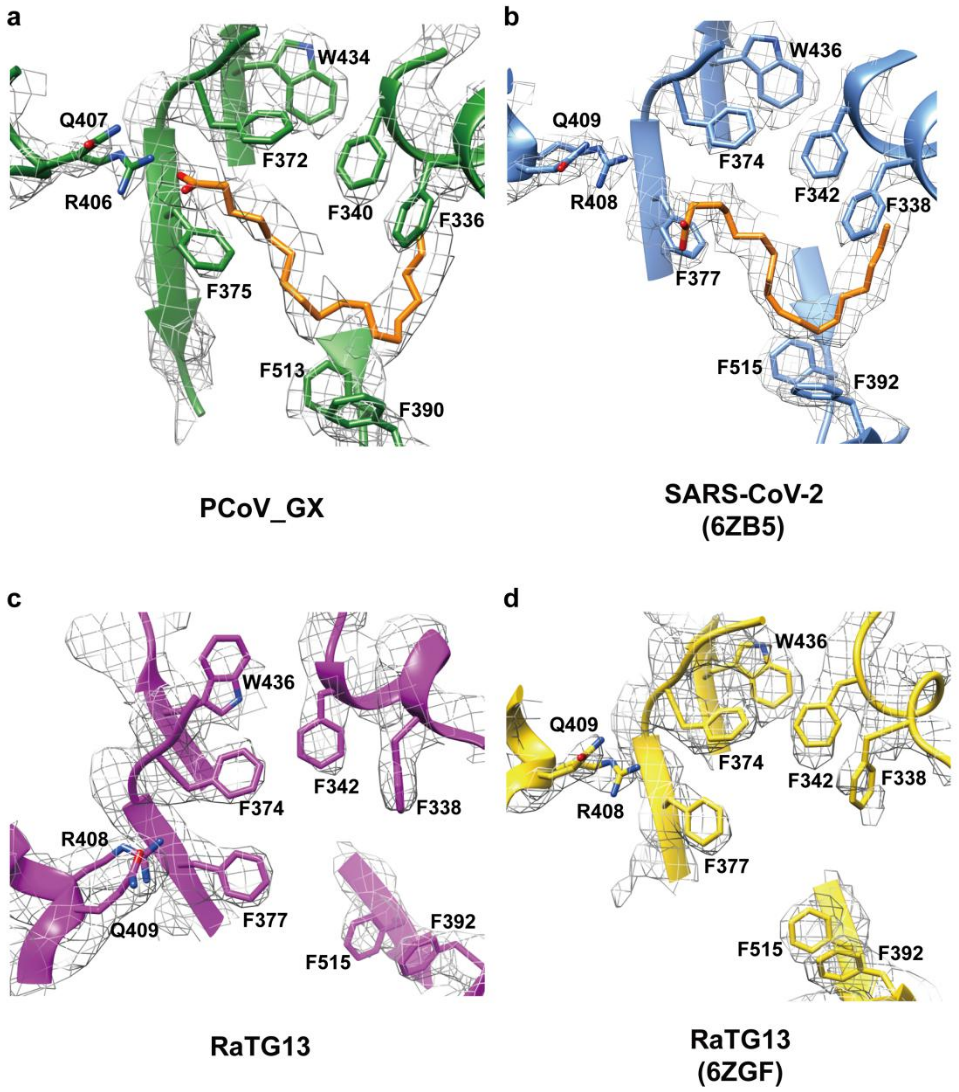

Supplementary Fig. 8 Binding of linoleic acid (LA) with the spikes. (**a**) LA-bound PCoV_GX spike. LA and the binding amino acid residues are shown as sticks with EM densities. LA is colored in orange. (**b**) LA-bound SARS-CoV-2 spike (PDB ID: 6ZB5). No densities of the LA were found in the same pocket of the RaTG13 spike structure determined by us (**c**) and by another group (**d**) (PDB ID: 6ZGF).

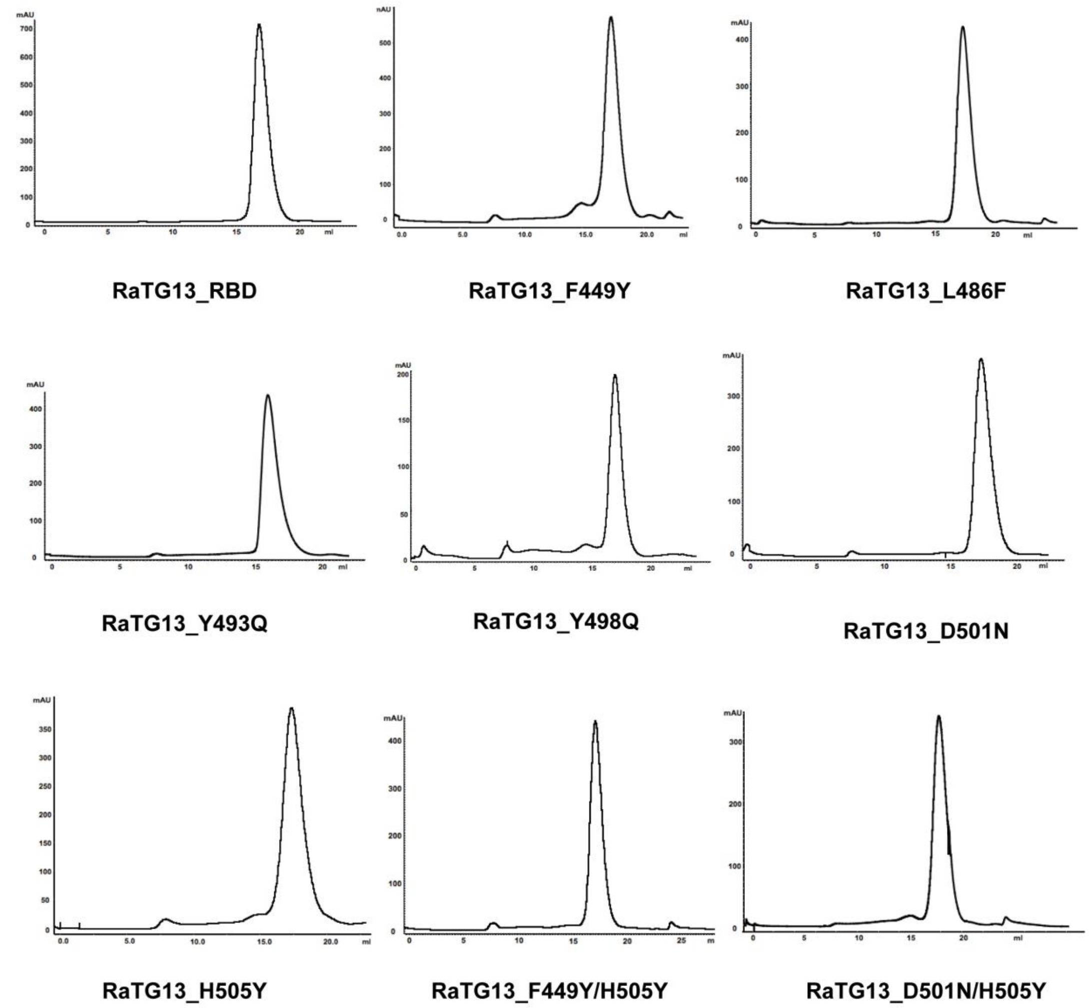

Supplementary Fig. 9 Purifications of the RaTG13 RBD and its mutants. Gel filtration chromatography of the RaTG13 RBD and its mutants.

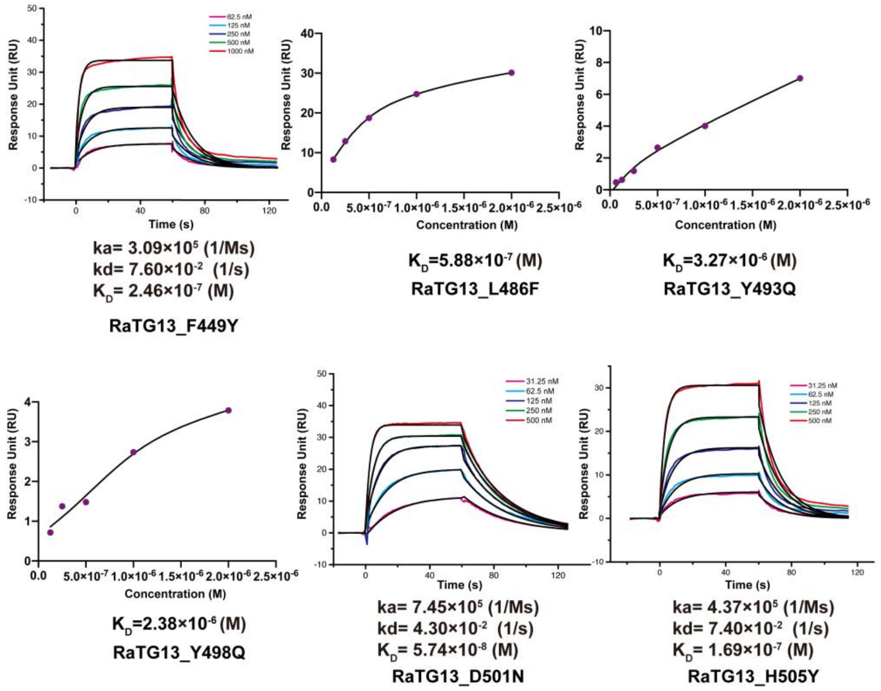

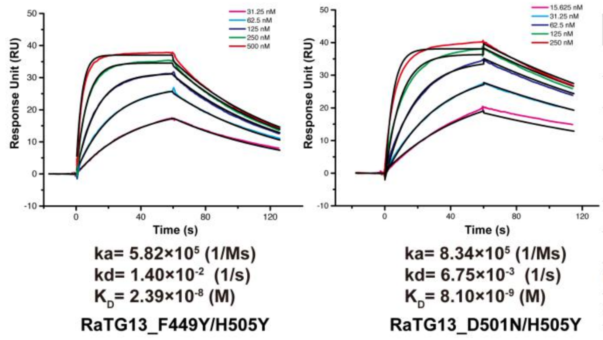

	K_D (nM)	Fold (K_D_RBD /K_D_mutants)
RaTG13_RBD	500	1
RaTG13_F449Y	246	2
RaTG13_L486F	588	1
RaTG13_Y493Q	3270	0.2
RaTG13_Y498Q	2380	0.2
RaTG13_D501N	57.4	9
RaTG13_H505Y	169	3
RaTG13_F449Y/H505Y	23.9	21
RaTG13_D501N/H505Y	8.1	62

Supplementary Fig. 10 Surface plasmon resonance sensorgrams and binding affinities of the RaTG13 RBD mutants with hACE2.

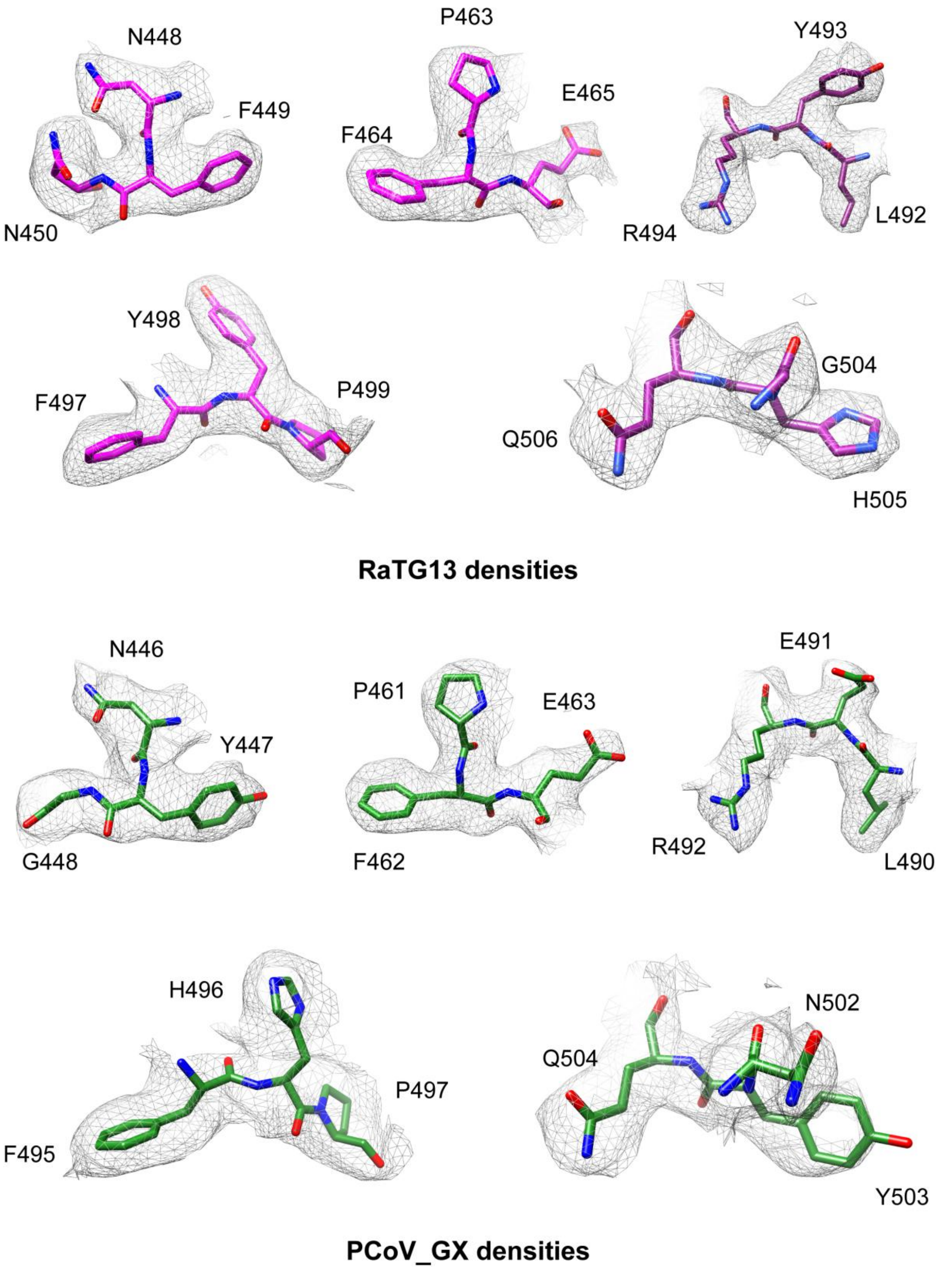

Supplementary Fig. 11 The representative density maps around amino acid residues in the RaTG13 and PCoV_GX RBDs. Each map is contoured at 2.5 RMS to show the density.

Supplementary Table 1. Cryo-EM data collection, refinement, and validation statistics

Data collection and processing	RaTG13 (PDB-7CN4 EMDB-30416)	PCoV_GX (PDB-7CN8 EMDB-30418)
Magnification	×81000	×81000
Voltage (kV)	300	300
Electron exposure (e–/Å^2)	50	50
Defocus range (μm)	-1.5 to -1.8	-1.5 to -1.8
Pixel size (Å)	1.0825	1.0825
Symmetry imposed	C3	C3
Initial particle images (no.)	~450,000	~700,000
Final particle images (no.)	99241	263842
Map resolution (Å)	2.93	2.48
FSC threshold	0.143	0.143
Map resolution range (Å)	2.93-6	2.48-5
Refinement		
Initial model used (PDB code)	5X58	6VSB
Model resolution (Å)	2.93	2.48
FSC threshold	0.143	0.143
Model resolution range (Å)	2.93	2.48
Map sharpening B factor (Å^2)	-30	-30
Model composition		
Non-hydrogen atoms	26253	27675
Protein residues	3360	3375
Ligands	54	96
B factors (Å^2)		
Protein	120.52	138.50
Ligand	98.97	106.49
R.m.s. deviations		
Bond lengths (Å)	0.007	0.013
Bond angles (°)	1.075	1.512
Validation		
MolProbity score	1.89	2.07
Clashscore	6.06	5.21
Poor rotamers (%)	1.32	4.85
Ramachandran plot		
Favored (%)	92.70	96.08
Allowed (%)	6.95	3.53

Disallowed (%)	0.36	0.39
Privateer		
Wrong anomer	0	0
Stereochemical problems	0	0
Unphysical puckering amplitude	0	0
In unlikely ring conformation	0	0

Supplementary Table 2. Amino acid residues and N-linked glycans interacting with the RBD in the RaTG13, PCoV_GX and SARS-CoV-2 spikes.

Spike	Monomer of counterclockwise	Monomer of clockwise	The number of interacting residues and glycans
RaTG13	41K, 43F, 113K, 115Q, 132E, 167T, 198D, 199G, 200Y, 230P, 231I, 232G, 234N, 369Y, 370N, 373S, 374F, 375S, 377F, 384P, 385T, 503V, 977L, 978S, 979R, 980L, 981D, 984E, N165-linked glycan, N234-linked glycan, N370-linked glycan	405D, 408R, 413G, 415T, 416G, 417K, 420D, 421Y, 455L, 503V, 505H, 982P, 983P	44
PCoV_GX	111R, 113Q, 163N, 196D, 197G, 198Y, 228P, 229I, 230G, 232N, 363Y, 367Y, 368N, 371S, 372F, 373S, 375F, 382P, 383T, 386N, 435N, 973D, 975L, 976S, 977R, 978L, 979D, N163-linked glycan, N232-linked glycan, N368-linked glycan	401K, 403D, 406R, 411G, 413T, 414G, 415V, 418D, 419Y, 451Y, 453L, 458K, 502N, 979D, 980P, 981P	46
SARS-CoV-2 (PDB: 6VXX)	41K, 200Y, 230P, 369Y, 370N, 979D, 981L, 982S, 983R, 984L, 985D, N234-linked glycan	415T,416G,417K, 421Y, 987P	17
SARS-CoV-2 (PDB: 6ZGE)	113K, 115Q, 198D, 199G, 200Y, 230P, 231I, 232G, 234N, 365Y, 369Y, 370N, 373S, 374F, 375S, 377F, 384P, 979D, 981L, 982S, 983R, 984L, 985D, 988E, N165-linked glycan, N234-linked glycan	403R, 405D, 408R, 413G, 415T, 416G, 417K, 420D, 421Y, 455L, 505Y	37

Supplementary Table 3. The DNA sequences of the spikes with codons optimization for protein purification

SARS-CoV-2 spike sequence (5'-3')
ATGTTCGTGTTCCTGGTGCTGCTGCCTCTGGTGAGCAGCCAGTGCGTGAATCTGACCACCAGAACCCAGCTGCCTCCTGCC<br>TACACCAATAGCTTCACCAGAGGAGTTTATTATCCCGATAAGGTGTTCAGAAGTAGTGTATTACATAGTACCCAGGACCTG<br>TTCCTACCTTTCTTCAGTAACGTGACCTGGTTCCACGCCATCCACGTGAGCGGCACCAATGGCACCAAGAGATTCGACAAT<br>CCTGTGCTGCCTTTCAATGACGGCGTGTACTTCGCCAGCACCGAGAAGAGCAATATCATCAGAGGCTGGATCTTCGGCACC<br>ACCTTGGATTCCAAGACTCAGAGCCTGCTGATTGTAAACAACGCTACAAATGTGGTGATCAAGGTGTGCGAGTTCCAGTTC<br>TGCAATGACCCTTTCCTGGGTGTTTATTATCATAAGAACAACAAGAGCTGGATGGAGAGCGAGTTCCGCGTATATTCGTCG<br>GCTAATAATTGCACCTTCGAGTACGTGAGCCAGCCTTTCCTGATGGACCTGGAGGGCAAGCAGGGCAATTTCAAGAATCTG<br>AGAGAGTTCGTGTTCAAGAATATCGACGGCTACTTCAAGATCTACAGCAAGCACACACCCATTAATCTGGTGAGAGACCT<br>GCCTCAGGGCTTCAGCGCCCTGGAGCCTCTGGTGGACCTGCCTATCGGCATCAATATCACCAGATTCCAGACCCTGCTGGC<br>CCTGCACAGATCATATCTTACACCAGGCGATTCGTCAAGCGGTTGGACCGCTGGAGCTGCGGCATATTACGTGGGCTACCT<br>GCAGCCTAGAACCTTCCTGCTGAAGTACAATGAGAATGGTACGATAACCGACGCAGTTGATTGTGCCCTGGACCCTCTGAG<br>CGAGACCAAGTGCACCCTGAAGAGCTTCACCGTGGAGAAGGGCATCTACCAGACCAGCAATTTCAGAGTGCAGCCTACCG<br>AGAGCATCGTGAGATTCCCTAATATCACCAATCTGTGCCCTTTCGGCGAGGTGTTCAATGCCACCAGATTCGCCAGCGTGT<br>ACGCATGGAACCGCAAGCGGATAAGCAATTGCGTGGCCGACTACAGCGTGCTGTACAATAGCGCCAGCTTCAGCACCTTC<br>AAATGTTATGGTGTTTCGCCAACAAAGCTGAATGACCTGTGCTTCACCAATGTGTACGCCGACAGCTTCGTGATCAGAGGC<br>GACGAGGTGAGACAGATCGCGCCAGGGCAGACCGGCAAGATCGCCGACTACAATTACAAGCTGCCTGACGACTTCACCGG<br>CTGCGTGATCGCGTGGAACTCTAACAATCTAGATTCGAAAGTTGGAGGCAATTACAATTACCTGTACAGACTGTTCAGAAA<br>GAGCAATCTGAAGCCTTTCGAGAGAGACATCAGCACCGAGATCTACCAGGCCGGCAGCACACCGTGTAATGGCGTGGAGG<br>GCTTCAATTGCTACTTCCCTCTGCAGAGCTACGGCTTCCAGCCTACCAATGGCGTGGGCTACCAGCCTTACAGAGTGGTGG<br>TGCTGAGCTTCGAGCTGCTGCACGCTCCCGCTACCGTGTGCGGCCCTAAGAAGAGCACCAATCTGGTGAAGAATAAGTGC<br>GTGAATTTCAATTTCAATGGTCTAACTGGAACGGGCGTGCTGACCGAGAGCAATAAGAAGTTTCTTCCCTTTCAACAATTC<br>GGCAGAGACATCGCCGACACCACAGATGCTGTAAGAGACCCTCAGACCCTGGAGATCCTGGACATCACTCCGTGTAGCTT<br>CGGCGGCGTGAGCGTGATCACACCGGGTACCAATACCAGCAATCAGGTGGCCGTGCTGTACCAGGACGTGAATTGCACCG<br>AGGTGCCTGTGGCCATCCACGCCGACCAGCTGACTCCCACTTGGAGGGTATATTCCACGGGAAGCAATGTGTTCCAGACCA<br>GAGCCGGCTGCCTGATCGGCGCCGAGCACGTGAATAATAGCTACGAGTGCGACATCCCTATCGGCGCCGGCATCTGCGCC<br>AGCTACCAGACCCAGACCAATAGCCCTAGAAGAGCCAGAAGCGTGGCCAGCCAGAGCATCATCGCCTACACCATGAGCCT<br>GGGCGCCGAGAATAGCGTGGCCTACAGCAATAATAGCATCGCCATCCCTACCAATTTCACCATCAGCGTGACCACCGAAA<br>TATTACCAGTCTCCATGACCAAGACCAGCGTGGACTGCACCATGTACATCTGCGGCGACAGCACCGAGTGCAGCAATCTG<br>CTGCTGCAGTACGGCAGCTTCTGCACCCAGCTGAATAGAGCCCTGACCGGCATCGCCGTGGAGCAGGACAAGAATACCCA<br>GGAGGTGTTCGCCCAGGTGAAGCAGATCTACAAGACTCCGCCGATCAAGGACTTCGGCGGCTTCAATTTCAGCCAAATAC<br>TCCCAGATCCAAGCAAGCCTAGCAAGAGGAGCTTCATCGAGGACCTGCTGTTCAATAAGGTGACCCTGGCCGACGCCGGC<br>TTCATCAAGCAGTACGGCGACTGCCTAGGTGATATTGCGGCAAGAGACCTGATCTGCGCCCAGAAGTTTAACGGTTTGACA<br>GTACTACCTCCTCTGCTGACCGACGAGATGATAGCACAATATACGTCGGCATTGCTCGCTGGCACGATCACATCGGGCTGG<br>ACTTTCGGCGCCGGAGCAGCGTTGCAAATCCCTTTCGCCATGCAGATGGCCTACAGATTCAATGGCATCGGCGTGACCCAG<br>AATGTGCTGTACGAGAATCAGAAGCTGATCGCCAATCAGTTCAATAGCGCCATCGGCAAGATCCAGGACAGCCTGAGCAG<br>CACCGCCAGCGCCCTGGGCAAGCTGCAGGACGTGGTGAATCAGAATGCCCAGGCCCTGAATACCCTGGTGAAGCAGCTGA<br>GCAGCAATTTCGGCGCCATCAGTAGTGTACTCAACGATATCCTGAGCAGACTGGACAAGGTGGAGGCCGAGGTGCAAATT<br>GATCGTCTTATTACTGGCAGACTGCAGAGCCTGCAGACCTACGTGACCCAGCAGCTGATCAGAGCCGCCGAGATCAGAGC<br>CAGCGCCAATCTGGCCGCCACCAAGATGAGCGAGTGCGTGCTGGGCCAGAGCAAGAGAGTGGACTTCTGCGGCAAGGGCT<br>ACCACCTGATGAGCTTCCCTCAGAGCGCTCCACATGGCGTGGTGTTCCTGCACGTGACCTACGTGCCTGCCCAGGAGAAGA

ATTTCACCACCGCACCCGCAATCTGCCACGACGGCAAGGCCCACTTCCCTAGAGAGGGCGTGTTCGTGAGCAATGGCACC<br>CACTGGTTCGTGACCCAGAGAAATTTCTACGAGCCTCAGATCATCACCACCGACAATACCTTCGTGAGCGGCAATTGCGAC<br>GTGGTGATCGGGATAGTCAATAATACTGTCTACGACCCTCTGCAGCCTGAGCTGGACAGCTTCAAGGAGGAGCTGGACAA<br>GTACTTCAAGAATCACACCAGCCCTGACGTGGACCTCGGTGATATTTCGGGAATCAATGCCAGCGTGGTGAATATCCAGA<br>AGGAAATTGATCGGCTCAACGAAGTGGCCAAGAATCTGAATGAGAGCCTGATCGACCTGCAGGAGCTGGGCAAGTACGA<br>GCAGTACATCAAG
RaTG13 spike sequence (5'-3')
ATGTTCGTGTTCCTGGTGCTGCTGCCCCTGGTGAGCAGCCAGTGCGTGAACCTGACCACCCGCACCCAGCTGCCCCCCGCC<br>TACACCAACAGCAGCACCCGCGGCGTGTACTACCCCGACAAGGTGTTCCGCAGCAGCGTGCTGCACCTGACCCAGGACCT<br>GTTCCTGCCCTTCTTCAGCAACGTGACCTGGTTCCACGCCATCCACGTGAGCGGCACCAACGGCATCAAGCGCTTCGACAA<br>CCCCGTGCTGCCCTTCAACGACGGCGTGTACTTCGCCAGCACCGAGAAGAGCAACATCATCCGCGGCTGGATCTTCGGCAC<br>CACCCTGGACAGCAAGACCCAGAGCCTGCTGATCGTGAACAACGCCACCAACGTGGTGATCAAGGTGTGCGAGTTCCAGT<br>TCTGCAACGACCCCTTCCTGGGCGTGTACTACCACAAGAACAACAAGAGCTGGATGGAGAGCGAGTTCCGCGTGTACAGC<br>AGCGCCAACAACTGCACCTTCGAGTACGTGAGCCAGCCCTTCCTGATGGACCTGGAGGGCAAGCAGGGCAACTTCAAGAA<br>CCTGCGCGAGTTCGTGTTCAAGAACATCGACGGCTACTTCAAGATCTACAGCAAGCACACCCCCATCAACCTGGTGCGCGA<br>CCTGCCCCCCGGCTTCAGCGCCCTGGAGCCCCTGGTGGACCTGCCCATCGGCATCAACATCACCCGCTTCCAGACCCTGCT<br>GGCCCTGCACCGCAGCTACCTGACCCCCGGCGACAGCAGCAGCGGCTGGACCGCCGGCGCCGCCGCCTACTACGTGGGCT<br>ACCTGCAGCCCCGCACCTTCCTGCTGAAGTACAACGAGAACGGCACCATCACCGACGCCGTGGACTGCGCCCTGGACCCC<br>CTGAGCGAGACCAAGTGCACCCTGAAGAGCTTCACCGTGGAGAAGGGCATCTACCAGACCAGCAACTTCCGCGTGCAGCC<br>CACCGACAGCATCGTGCGCTTCCCCAACATCACCAACCTGTGCCCCTTCGGCGAGGTGTTCAACGCCACCACCTTCGCCAG<br>CGTGTACGCCTGGAACCGCAAGCGCATCAGCAACTGCGTGGCCGACTACAGCGTGCTGTACAACAGCACCAGCTTCAGCA<br>CCTTCAAGTGCTACGGCGTGAGCCCCACCAAGCTGAACGACCTGTGCTTCACCAACGTGTACGCCGACAGCTTCGTGATCA<br>CCGGCGACGAGGTGCGCCAGATCGCCCCCGGCCAGACCGGCAAGATCGCCGACTACAACTACAAGCTGCCCGACGACTTC<br>ACCGGCTGCGTGATCGCCTGGAACAGCAAGCACATCGACGCCAAGGAGGGCGGCAACTTCAACTACCTGTACCGCCTGTT<br>CCGCAAGGCCAACCTGAAGCCCTTCGAGCGCGACATCAGCACCGAGATCTACCAGGCCGGCAGCAAGCCCTGCAACGGCC<br>AGACCGGCCTGAACTGCTACTACCCCCTGTACCGCTACGGCTTCTACCCCACCGACGGCGTGGGCCACCAGCCCTACCGCG<br>TGGTGGTGCTGAGCTTCGAGCTGCTGAACGCCCCCGCCACCGTGTGCGGCCCCAAGAAGAGCACCAACCTGGTGAAGAAC<br>AAGTGCGTGAACTTCAACTTCAACGGCCTGACCGGCACCGGCGTGCTGACCGAGAGCAACAAGAAGTTCCTGCCCTTCCA<br>GCAGTTCGGCCGCGACATCGCCGACACCACCGACGCCGTGCGCGACCCCCAGACCCTGGAGATCCTGGACATCACCCCCT<br>GCAGCTTCGGCGGCGTGAGCGTGATCACCCCCGGCACCAACGCCAGCAACCAGGTGGCCGTGCTGTACCAGGACGTGAAC<br>TGCACCGAGGTGCCCGTGGCCATCCACGCCGACCAGCTGACCCCCACCTGGCGCGTGTACAGCACCGGCAGCAACGTGTT<br>CCAGACCCGCGCCGGCTGCCTGATCGGCGCCGAGCACGTGAACAACAGCTACGAGTGCGACATCCCCATCGGCGCCGGCA<br>TCTGCGCCAGCTACCAGACCCAGACCAACAGCCGCAGCGTGGCCAGCCAGAGCATCATCGCCTACACCATGAGCCTGGGC<br>GCCGAGAACAGCGTGGCCTACAGCAACAACAGCATCGCCATCCCCACCAACTTCACCATCAGCGTGACCACCGAGATCCT<br>GCCCGTGAGCATGACCAAGACCAGCGTGGACTGCACCATGTACATCTGCGGCGACAGCACCGAGTGCAGCAACCTGCTGC<br>TGCAGTACGGCAGCTTCTGCACCCAGCTGAACCGCGCCCTGACCGGCATCGCCGTGGAGCAGGACAAGAACACCCAGGAG<br>GTGTTCGCCCAGGTGAAGCAGATCTACAAGACCCCCCCCATCAAGGACTTCGGCGGCTTCAACTTCAGCCAGATCCTGCCC<br>GACCCCAGCAAGCCCAGCAAGCGCAGCTTCATCGAGGACCTGCTGTTCAACAAGGTGACCCTGGCCGACGCCGGCTTCAT<br>CAAGCAGTACGGCGACTGCCTGGGCGACATCGCCGCCCGCGACCTGATCTGCGCCCAGAAGTTCAACGGCCTGACCGTGC<br>TGCCCCCCCTGCTGACCGACGAGATGATCGCCCAGTACACCAGCGCCCTGCTGGCCGGCACCATCACCAGCGGCTGGACCT<br>TCGGCGCCGGCGCCGCCCTGCAGATCCCCTTCGCCATGCAGATGGCCTACCGCTTCAACGGCATCGGCGTGACCCAGAACG<br>TGCTGTACGAGAACCAGAAGCTGATCGCCAACCAGTTCAACAGCGCCATCGGCAAGATCCAGGACAGCCTGAGCAGCACC<br>GCCAGCGCCCTGGGCAAGCTGCAGGACGTGGTGAACCAGAACGCCCAGGCCCTGAACACCCTGGTGAAGCAGCTGAGCA<br>GCAACTTCGGCGCCATCAGCAGCGTGCTGAACGACATCCTGAGCCGCCTGGACAAGGTGGAGGCCGAGGTGCAGATCGAC

CGCCTGATCACCGGCCGCCTGCAGAGCCTGCAGACCTACGTGACCCAGCAGCTGATCCGCGCCGCCGAGATCCGCGCCAG<br>CGCCAACCTGGCCGCCACCAAGATGAGCGAGTGCGTGCTGGGCCAGAGCAAGCGCGTGGACTTCTGCGGCAAGGGCTACC<br>ACCTGATGAGCTTCCCCCAGAGCGCCCCCCACGGCGTGGTGTTCCTGCACGTGACCTACGTGCCCGCCCAGGAGAAGAACT<br>TCACCACCGCCCCCGCCATCTGCCACGACGGCAAGGCCCACTTCCCCCGCGAGGGCGTGTTCGTGAGCAACGGCACCCACT<br>GGTTCGTGACCCAGCGCAACTTCTACGAGCCCCAGATCATCACCACCGACAACACCTTCGTGAGCGGCAGCTGCGACGTG<br>GTGATCGGCATCGTGAACAACACCGTGTACGACCCCCTGCAGCCCGAGCTGGACAGCTTCAAGGAGGAGCTGGACAAGTA<br>CTTCAAGAACCACACCAGCCCCGACGTGGACCTGGGCGACATCAGCGGCATCAACGCCAGCGTGGTGAACATCCAGAAGG<br>AGATCGACCGCCTGAACGAGGTGGCCAAGAACCTGAACGAGAGCCTGATCGACCTGCAGGAGCTGGGCAAGTACGAGCA<br>GTACATCAAGTGGCCC
PCoV_GX spike sequence (5'-3')
ATGTTTGTGTTCCTATTCGTCCTACCTCTTGTGTCATCACAATGCGTGAACCTTACAACAAGAACAGGAATCCCTCCTGGAT<br>ACACAAACTCATCAACAAGAGGAGTGTACTACCCTGACAAGGTGTTTAGATCATCAATCCTTCACCTTACACAAGATCTCT<br>TTCTACCGTTCTTCTCGAACGTGACATGGTTTAACACAATCAACTACCAAGGAGGATTTAAGAAGTTCGACAACCCTGTGC<br>TTCCTTTCAATGACGGAGTGTACTTTGCATCAACAGAGAAGTCTAATATCATCAGAGGATGGATCTTTGGAACAACACTTG<br>ATGCAAGAACACAATCACTTCTTATTGTCAATAATGCTACGAACGTGGTGATCAAAGTGTGCGAATTTCAATTCTGTACTG<br>ATCCTTTCCTAGGCGTTTACTACCACAACAACAACAAGACCTGGGTGGAGAATGAGTTTCGTGTATATAGCTCGGCGAACA<br>ACTGCACATTTGAATACATCTCACAACCTTTCTTAATGGATCTTGAAGGAAAGCAGGGTAACTTTAAAAACCTTCGTGAAT<br>TTGTGTTTAAGAATGTCGATGGATACTTTAAGATATATTCAAAGCATACTCCAATCGACTTGGTTCGGGATCTTCCTAGAG<br>GATTTGCAGCACTTGAACCTCTTGTGGATCTTCCTATCGGAATCAACATCACAAGATTTCAAACACTTCTTGCACTTCACAG<br>ATCATACCTTACACCTGGAAACCTTGAATCTGGCTGGACCACGGGCGCAGCAGCATACTACGTGGGATACCTTCAACAAA<br>GAACATTTCTTCTTTCATACAACCAGAATGGAACGATTACAGATGCGGTCGACTGTTCACTTGATCCTCTTTCAGAAACAA<br>AGTGTACTCTTAAATCACTTACAGTGGAGAAGGGTATTTACCAAACATCAAACTTTAGAGTGCAACCTACAATCTCAATCG<br>TGAGATTTCCTAACATCACAAACCTTTGCCCTTTCGGTGAAGTCTTTAACGCATCAAAGTTCGCGTCAGTGTACGCATGGA<br>ACAGAAAGCGCATTTCAAACTGCGTGGCAGATTACTCAGTGCTTTACAACTCAACATCATTCAGCACCTTTAAATGCTACG<br>GAGTGTCACCTACAAAGTTAAATGATCTTTGCTTTACAAACGTGTACGCAGATTCATTTGTGGTGAAAGGAGATGAAGTGA<br>GACAAATCGCACCTGGACAAACAGGAGTGATCGCAGATTACAACTACAAACTTCCTGATGATTTCACTGGGTGCGTGATC<br>GCATGGAACTCAGTGAAACAAGATGCCCTGACTGGTGGCAACTATGGTTATTTATATCGCCTCTTTCGGAAGAGTAAGCTC<br>AAACCTTTCGAGCGGGACATAAGCACCGAGATCTACCAAGCAGGATCAACACCTTGCAACGGACAAGTGGGACTTAACTG<br>CTACTACCCTCTTGAAAGATACGGATTTCACCCTACAACAGGAGTGAACTACCAACCTTTCCGTGTCGTGGTGCTTTCATTT<br>GAACTTCTTAACGGACCTGCAACAGTGTGCGGACCTAAACTTTCAACAACGCTCGTTAAGGACAAGTGCGTGAACTTTAAC<br>TTCAATGGTTTGACGGGTACTGGAGTGCTTACAACATCAAAGAAGCAGTTTCTCCCATTCCAGCAATTTGGACGCGATATC<br>TCAGATACGACGGACGCCGTTCGAGACCCTCAAACACTTGAAATCCTTGATATCACACCTTGCTCATTTGGAGGAGTGTCA<br>GTGATCACACCTGGAACAAACACATCAAACCAAGTGGCAGTGCTTTACCAAGATGTGAACTGCACAGAAGTGCCTATGGC<br>AATCCACGCAGAACAACTTACACCTGCATGGCGCGTATATTCGGCTGGTGCAAACGTGTTTCAAACAAGAGCAGGATGCC<br>TTGTGGGAGCAGAACACGTGAACAACTCATACGAATGCGATATCCCTGTGGGAGCAGGAATCTGCGCATCATACCACTCA<br>ATGTCATCACTTAGATCAGTGAACCAAAGATCAATCATCGCATACACAATGTCACTTGGAGCAGAAAACTCAGTGGCATA<br>CTCAAACAACTCAATCGCAATCCCTACAAACTTTACAATCTCAGTGACAACAGAGATTCTCCCAGTGTCAATGACAAAGAC<br>TTCCGTGGATTGCACAATGTACATCTGCGGAGATTCAATCGAATGCTCAAACCTTCTTCTTCAATACGGATCATTCTGTACT<br>CAACTTAACAGAGCCCTAACGGGCATAGCTGTGGAACAAGATAAGAATACGCAAGAAGTGTTTGCACAAGTGAAACAAA<br>TCTACAAGACTCCGCCTATCAAAGATTTCGGCGGTTTCAATTTCAGTCAGATATTACCAGACCCTAGCAAACCGAGCAAGC<br>GCTCATTTATCGAAGATCTGCTCTTTAATAAGGTGACACTTGCAGATGCAGGATTTATCAAACAATACGGAGATTGCCTCG<br>GCGACATAGCCGCCAGAGATCTTATCTGCGCACAGAAGTTTAACGGCCTGACCGTATTGCCTCCTCTTCTTACAGATGAAA<br>TGATCGCACAATACACATCAGCACTTCTTGCAGGGACGATAACTAGCGGATGGACTTTCGGTGCGGGCGCAGCACTTCAA<br>ATCCCTTTCGCGATGCAAATGGCATACAGATTTAACGGAATCGGAGTGACACAGAATGTACTTTACGAGAATCAGAAACT

CATTGCAAACCAATTTAACTCAGCAATCGGAAAGATTCAGGATTCACTTTCATCAACAGCATCAGCACTTGGAAAGCTCCA
GGATGTGGTGAACCAGAATGCGCAAGCACTTAACACGTTGGTTAAGCAGCTATCATCAAACTTTGGAGCAATCTCATCAGT
GCTTAACGATATCCTTTCAAGACTTGATAAAGTGGAAGCAGAAGTGCAGATTGACCGTCTAATTACGGGAAGACTTCAATC
ACTTCAAACATACGTTACCCAGCAGTTAATAAGAGCAGCAGAAATCAGAGCATCAGCAAACCTTGCAGCAACAAAGATGT
CGGAATGCGTGCTTGGACAATCAAAGAGGGTAGATTTCTGTGGCAAGGGCTACCATCTTATGTCATTTCCTCAATCAGCAC
CTCACGGAGTGGTGTTTCTTCACGTGACATACGTGCCTGCACAAGAGAAGAATTTCACAACAGCACCTGCAATCTGCCACG
AAGGAAAGGCTCATTTCCCGCGAGAAGGAGTGTTTGTGTCAAACGGAACACACTGGTTTATCACACAAAGAAACTTCTAT
GAGCCTCAAATCATCACAACAGATAACACATTTGTGTCAGGATCATGCGATGTGGTGATCGGAATAGTTAACAACACAGT
ATATGACCCTCTTCAACCTGAACTTGATTCATTTAAAGAAGAACTTGATAAATACTTTAAAAACCACACATCACCTGATGT
GGATTTGGGAGATATTTCAGGGATAAACGCATCAGTGGTGAACATCCAGAAGGAAATCGACCGACTCAATGAGGTGGCAA
AGAATCTAAACGAAAGCCCGATTGACCTCCAGGAGCTTGGAAAGTATGAGCAATACATCAAA

Supplementary Table 4. Primers used in this manuscript.

Name	Sequence (5'-3')
RaTG13-spike-F-pCAG	TTATCGATCCGGAACGCGTCTCGAGGAATTCATGTTCGTGTTCCTGGTGC
RaTG13-spike-R-pCAG	CCAGCCCTGAAAATACAGGTTTTCGGTACCTCACTTTTCAAACTGGGGGT
PCoV_GX-spike-F-pCAG	TATCGATCCGGAACGCGTCTCGAGGAATTCATGTTTGTGTTCCTATTCGT
PCoV_GX-spike-R-pCAG	CCAGCCCTGAAAATACAGGTTTTCGGTACCTCACTTTTCAAACTGGGGGT
RaTG13-spike-T372A-F-pCAG	TACAGCGTGCTGTACAACAGCGCCAGCTTCAGCACCTTCAAGTGC
RaTG13-spike-T372A-R-pCAG	GCACTTGAAGGTGCTGAAGCTGGCGCTGTTGTACAGCACGCTGTA
PCoV_GX-spike-T370A-F-pCAG	TACTCAGTGCTTTACAACTCAGCATCATTCAGCACCTTTAAATGC
PCoV_GX-spike-T370A-R-pCAG	GCATTTAAAGGTGCTGAATGATGCTGAGTTGTAAAGCACTGAGTA
SARS-CoV-2-RBD-333-F-B12	CTGCCTTTGCGGCGGATCCACCAATCTGTGCCCTTTC
SARS-CoV-2-RBD-527-R-B12	TAGTACTTCTCGACAAGCTTCTAATGGTGATGGTGATGGTGAGG
PCoV_GX-RBD-331-F-B12	CTGCCTTTGCGGCGGATCCACAAACCTTTGCCCTTTC
PCoV_GX-RBD-524-R-B12	TAGTACTTCTCGACAAGCTTCTAATGGTGATGGTGATGGTGTC
RaTG13-RBD-333-F-B12	CTGCCTTTGCGGCGGATCCACCAACCTGTGCCCCTTC
RaTG13-RBD-526-R-B12	TAGTACTTCTCGACAAGCTTCTAATGGTGATGGTGATGGTGG
RaTG13-RBD-333-F-pCAG	ATTCCACGCGTCTCGAGGAATTCACCAACCTGTGCCCCTTCG
RaTG13-RBD-526-R-pCAG	GAAAATACAGGTTTTCGGTACCCTAATGGTGATGGTGATGGTGGCCGC
RaTG13-F449Y-F-pCAG	AAGGAGGGCGGCAACTACAACTACCTGTACCGCCTGTTC
RaTG13-F449Y-R-pCAG	GAACAGGCGGTACAGGTAGTTGTAGTTGCCGCCCTCCTT
RaTG13-L486F-F-pCAG	TGCAACGGCCAGACCGGCTTCAACTGCTACTACCCCCTG
RaTG13-L486F-R-pCAG	CAGGGGGTAGTAGCAGTTGAAGCCGGTCTGGCCGTTGCA
RaTG13-Y493Q-F-pCAG	CTACTACCCCCTGCAGCGCTACGGCTTCTACCCCACC
RaTG13-Y493Q-R-pCAG	GGTGGGGTAGAAGCCGTAGCGCTGCAGGGGGTAGTAG
RaTG13-Y498Q-F-pCAG	CGCTACGGCTTCCAGCCCACCGACGGCGTGGGC
RaTG13-Y498Q-R-pCAG	GCCCACGCCGTCGGTGGGCTGGAAGCCGTAGCG
RaTG13-D501N-F-pCAG	TTCTACCCCACCAACGGCGTGGGCCACCAGCCC
RaTG13-D501N-R-pCAG	GGGCTGGTGGCCCACGCCGTTGGTGGGGTAGAA
RaTG13-H505Y-F-pCAG	GACGGCGTGGGCTACCAGCCCTACCGCGTGG
RaTG13-H505Y-R-pCAG	CCACGCGGTAGGGCTGGTAGCCCACGCCGTC
