# Supplementary material for: Bat and pangolin coronavirus spike glycoprotein structures provide insights into SARS-CoV-2 evolution
Source: Nat Commun. 2021 Mar 11;12:1607. doi: 10.1038/s41467-021-21767-3 (PMC7952905; doi:10.1038/s41467-021-21767-3)
Supplement: Supplementary file 2 — Reporting Summary [file 41467_2021_21767_MOESM2_ESM.pdf]

nature research

Corresponding author(s): Xinquan Wang

Last updated by author(s): Feb 5, 2021

Reporting Summary

Nature Research wishes to improve the reproducibility of the work that we publish. This form provides structure for consistency and transparency in reporting. For further information on Nature Research policies, see our Editorial Policies and the Editorial Policy Checklist.

Statistics

For all statistical analyses, confirm that the following items are present in the figure legend, table legend, main text, or Methods section.

n/a	Confirmed	
☐	☒	The exact sample size (*n*) for each experimental group/condition, given as a discrete number and unit of measurement
☒	☐	A statement on whether measurements were taken from distinct samples or whether the same sample was measured repeatedly
☒	☐	The statistical test(s) used AND whether they are one- or two-sided<br>*Only common tests should be described solely by name; describe more complex techniques in the Methods section.*
☒	☐	A description of all covariates tested
☒	☐	A description of any assumptions or corrections, such as tests of normality and adjustment for multiple comparisons
☐	☒	A full description of the statistical parameters including central tendency (e.g. means) or other basic estimates (e.g. regression coefficient) AND variation (e.g. standard deviation) or associated estimates of uncertainty (e.g. confidence intervals)
☒	☐	For null hypothesis testing, the test statistic (e.g. *F*, *t*, *r*) with confidence intervals, effect sizes, degrees of freedom and *P* value noted<br>*Give P values as exact values whenever suitable.*
☒	☐	For Bayesian analysis, information on the choice of priors and Markov chain Monte Carlo settings
☒	☐	For hierarchical and complex designs, identification of the appropriate level for tests and full reporting of outcomes
☒	☐	Estimates of effect sizes (e.g. Cohen's *d*, Pearson's *r*), indicating how they were calculated

Our web collection on statistics for biologists contains articles on many of the points above.

Software and code

Policy information about availability of computer code

Data collection	There is no open source software for collecting data.
Data analysis	The AutoEmation2 is a collecting software but is not open source. RELION-3.1, MotionCor2 v.1.2.6, GCTF v.1.18, EMAN2, Gautomatch v.0.56, Resmap1.1.4, Coot v.0.9.2 and PHENIX v.1.18.2 were used at the determination of the structure for data processing , model building and refinement. Privateer was used for glycan validation. PyMOL 2.0 and UCSF Chimera v.1.15 was used to generate the structural figures. GraphPad Prism8 and Biacore T200 Evaluation Software 3.1 were used to do data analysis and make some of the figures.

For manuscripts utilizing custom algorithms or software that are central to the research but not yet described in published literature, software must be made available to editors and reviewers. We strongly encourage code deposition in a community repository (e.g. GitHub). See the Nature Research guidelines for submitting code & software for further information.

Data

Policy information about availability of data

All manuscripts must include a data availability statement. This statement should provide the following information, where applicable:

- Accession codes, unique identifiers, or web links for publicly available datasets
- A list of figures that have associated raw data
- A description of any restrictions on data availability

Cryo-EM structures presented in this work have been deposited in the Protein Data Bank (PDB) under the accession codes 7CN8 for the PCoV_GX spike and 7CN4 for the RaTG13 spike. The maps have been deposited into the Electron Microscopy Data Bank (EMDB) under codes EMD-30418 for the PCoV_GX spike and EMD-30416 for the RaTG13 spike. And the other structures we used in the study for analysis, PDB ID: 6M0J, 6VXX, 6ZGE, 6ZGF, 6ZB5, were searched from the Protein Data Bank (PDB). The source data underlying Figs. 5–6 and Supplementary Figs. 1, 10 are provided as a Source Data file with this paper. Any other raw data pertaining to this study are available from the corresponding author upon reasonable request.

Field-specific reporting

Please select the one below that is the best fit for your research. If you are not sure, read the appropriate sections before making your selection.

- [x] Life sciences
- [ ] Behavioural & social sciences
- [ ] Ecological, evolutionary & environmental sciences

For a reference copy of the document with all sections, see nature.com/documents/nr-reporting-summary-flat.pdf

Life sciences study design

All studies must disclose on these points even when the disclosure is negative.

Sample size	3963 images of PCoV_GX spike and 1889 images of RaTG13 spike were collected using FEI Titan Krios microscope. The number of images were sufficient to achieve the reported resolution in cryoEM.
Data exclusions	Cryo-EM single particles were included and excluded using standard data processing, as detailed in Supplementary Figure 2.
Replication	The mass spectrometry analysis were performed two times independently. And the pseudovirus entry assays were performed three times independently. All attempts at replication were successful. The cryoEM data collection was performed once and structures were determined from independent half datasets, which were compared to assess the resolution of the reconstruction. Both half-set replicates were the same to the stated resolution.
Randomization	There is no allocation in this study, so randomization is not applicable.
Blinding	No blinding was conducted since there was no specific grouping in data collection and analysis.

Reporting for specific materials, systems and methods

We require information from authors about some types of materials, experimental systems and methods used in many studies. Here, indicate whether each material, system or method listed is relevant to your study. If you are not sure if a list item applies to your research, read the appropriate section before selecting a response.

Materials & experimental systems

n/a	Involved in the study	
x		Antibodies
	x	Eukaryotic cell lines
x		Palaeontology and archaeology
x		Animals and other organisms
x		Human research participants
x		Clinical data
x		Dual use research of concern

Methods

n/a	Involved in the study	
x		ChIP-seq
x		Flow cytometry
x		MRI-based neuroimaging

Eukaryotic cell lines

Policy information about cell lines

Cell line source(s)	SF9, Hi5, FreeStyle 293-F, HEK 293T cells were bought from ATCC.
Authentication	All cell lines were frequently checked for cellular morphologies, growth rates and functions. All cell lines were available in commercial company.
Mycoplasma contamination	We confirm that all cell lines were negative for mycoplasma contamination.
Commonly misidentified lines (See ICLAC register)	No commonly misidentified cell lines were used.
